# Sex-specific dietary habits and their association with weight change in healthy adults

**DOI:** 10.1186/s12916-024-03730-3

**Published:** 2024-11-06

**Authors:** Michal Rein, Matan Elkan, Anastasia Godneva, Noa Cohen Dolev, Eran Segal

**Affiliations:** 1 Department of Computer Science and Applied Mathematics, The Weizmann Institute of Science, Rehovot, Israel; 2 Department of Molecular Cell Biology, The Weizmann Institute of Science, Rehovot, Israel; 3 Department of Internal Medicine A, Shamir Medical Center (Assaf Harofeh), Zerifin, Israel

**Keywords:** Body mass index, Sex, Diet, Weight loss, Nutritional epidemiology, Personalized nutrition

## Abstract

**Background:**

Dietary intake plays a pivotal role in the prevalence and management of obesity. While women and men exhibit differences in dietary habits and food-related behaviors, sex-based weight loss recommendations are lacking. This study aims to examine the impact of specific foods and food categories on weight reduction in men and women over a two-year period.

**Methods:**

A total of 8,548 participants from the 10K cohort, from 2019 to 2023, were included in the analysis (53.1% women, mean age 51.7 years). Anthropometric measurements and laboratory results were collected at baseline and at the two-year follow-up visit. Dietary assessment was based on daily food intake digitally logged through an application for at least 3 consecutive days at both timepoints. We compared intake of macronutrients, micronutrients, food groups and daily energy consumption between sex and body mass index (BMI) categories at baseline and weight change categories at follow-up. Using linear regression, we assessed the associations between food categories or specific foods and BMI at baseline as well as weight change percentage at follow-up.

**Results:**

Dietary habits varied by BMI and sex. Women and men living with obesity (BMI > 30 kg/m^2^) reported a greater intake of animal-based protein and lower intake of plant-based proteins and fats at baseline, as compared to participants with normal weight. In linear regression models predicting two-year weight change, including age, income, and baseline weight, the explained variance was 5.6% for men and 5.8% for women. Adding food categories and specific foods increased the explained variance to 20.6% for men and 17.5% for women. Weight reduction in men was linked to daily consumption of an egg (1.2% decrease) and beef (1.5% decrease), while in women, the most pronounced reductions were associated with an apple (1.2% decrease) and cashew nuts (3.4% decrease). Notably, total energy intake changes significantly impacted weight outcomes only in women.

**Conclusions:**

Sex-specific dietary habits significantly influence weight change over time. In men, weight loss was primarily associated with the addition of animal-based protein, while in women, it was linked to caloric deficit and plant-based fat, suggesting that sex-based nutritional interventions may demonstrate greater efficacy.

**Trial registration:**

NCT05817734 (retrospectively registered January 31, 2023).

**Supplementary Information:**

The online version contains supplementary material available at 10.1186/s12916-024-03730-3.

## Background

Excess weight and obesity pose significant risks for various health complications, such as cardiovascular disease, diabetes, and certain cancers [1–3]. In recent decades, there has been a marked increase in global obesity rates [4, 5], impacting both sexes. Global estimates in 2022 indicated that 43% of men and 44% of women were overweight, while 14% of men and 18% of women were living with obesity [6]. The general population of Israel reflects this upward trend, with 43% of men and 33.7% of women being overweight, and 19% of men and 17% of women living with obesity [6, 7].

Dietary intake plays a pivotal role in the prevalence and management of obesity. Factors such as daily energy consumption, macronutrient distribution, and food quality are closely linked to obesity and associated clinical risks [8, 9]. For example, dietary habits rich in vegetables, fruits, nuts, whole grains, unsaturated vegetable oils, and fish along with limited intake of processed meat, high-fat dairy, and refined carbohydrates or sweets have been associated with a decreased risk of all-cause mortality [1]. Not surprisingly, most of these foods were found to be associated with weight changes in both sexes [10]. Nonetheless, the relationship between diet and obesity remains a subject of ongoing debate [11], including the role of dietary habits on sex-based differences in obesity rates and general health [12].

Studies have indicated noticeable differences between men and women regarding macronutrient intake and adherence to dietary recommendations [8–10], in addition to factors such as age, education, and income. Typically, women exhibit a greater inclination toward health-conscious behaviors and report healthier diets than men [13]. Furthermore, they tend to prefer higher-quality foods, such as fruits and vegetables [13, 14]. However, while women generally consume fewer calories than men, when adjusting for weight, their energy and macronutrient intake often surpasses the recommended levels compared to men [10].

According to an Israeli national survey (MABAT), which assessed health status, dietary habits, and nutritional status, men reported greater energy intake than women, despite both sexes having comparable macronutrient compositions [15]. However, these findings, based on dietary questionnaires, are limited by potential inaccuracies in dietary assessment and the induction period required to observe diet-disease associations [16]. More accurate data is essential for fully understanding population dietary habits and establishing sex-specific dietary guidelines for weight management.

In this study, we provide a detailed description of the actual dietary habits of 10K Project participants [17], employing an advanced digital data collection method for enhanced accuracy and quality of the data [18–20]. Our objective was to explore the relationships among dietary habits, body mass index (BMI), and weight changes, offering insights into the dynamics between diet and weight management.

## Methods

### Study design and population

This study was conducted as part of the nation-wide 10K Project study, the full details of which have been previously described elsewhere [17]. Briefly, this ongoing project involves a large cohort of healthy adult participants with deep multi-omics profiling and long-term follow-up, including onsite meetings every two years. Registration for the study began on October 28, 2018, with participants aged between 40 and 70 years at baseline. The novelty of the project is the combination of innovative medical tests and advanced artificial intelligence methods to discover personal characteristics that can help predict future medical conditions, even before they manifest. The 10K Project is conducted according to the principles of the Declaration of Helsinki and was approved by the Institutional Review Board of the Weizmann Institute of Science.

At the time of this study, 9,988 individuals had been recruited for the 10K Project, and a baseline visit was made. Of these, 8,548 participants had adequate dietary data recorded, with inclusion criteria requiring three days or more of logged 500 to 4000 kcal/day. Furthermore, 1,961 of these participants attended an onsite visit for a two-year follow-up and had subsequent dietary recordings. Six participants were excluded due to a change in weight beyond 25% since it might represent an underlying illness or pathological process.

### Dietary assessment

Participants in the 10k Project were instructed to log their food intake in real time over a two-week period during each visit using a designated smartphone app (“Project 10K app”). In total, dietary data included 394,801 days of logging, with a median of 17 days and 1585 ± 606 kcal per day per participant.  This app, specifically developed for the cohort, features a database of more than 7,000 foods with full nutritional value and is based on the Israeli Ministry of Health database, which was further expanded with additional items from certified sources. Participants select each food item from the database, noting its weight or portion size, and log it into their user profile. Notably, the application has been employed in multiple studies over the past decade [19–21]. The logging data underwent a quality control process, including removing items with improbable weights or improbable timing (e.g., many meals logged within a short time period). Moreover, the dietary data has been correlated with serum metabolites linked to diet, providing an objective validation [22].

Our analyses encompassed 21 macronutrients and micronutrients and 34 distinct food categories. Macronutrients included carbohydrates, protein, and total dietary fat. Values were computed as the absolute amount consumed in grams per day as well as the percentage of total daily energy. Total dietary fiber was evaluated as grams per 1000 kcal consumed per day. Subtypes of dietary fats, including saturated fatty acids (SatFat), monounsaturated fatty acids (MUFAs), and polyunsaturated fatty acids (PUFAs), were computed by weight. The micronutrients included: cholesterol, calcium, magnesium, iron, potassium, sodium, vitamin A (RAE), vitamin B1 (thiamin), vitamin B3 (niacin), vitamin B6 (pyridoxine), vitamin B9 (total folate), vitamin B12, vitamin C, and vitamin E. These values were computed as absolute daily consumption (mg or µg). Certain micronutrients with negligible counts or inconsistent annotations in the food database were excluded from our analysis (e.g., vitamin K, vitamin B7 (biotin), iodine, and trans fatty acids).

All food items logged by participants in the mobile app (with more than 10 counts) were classified into 34 common food categories based on their botanical and nutritional properties (Supplementary Material 1: Table S1-S2). The average daily energy intake was used to evaluate consumption within these categories. Importantly, from a practical perspective, we computed both the energy intake from each food category and its proportion of the total daily intake. Specifically, the category of ultra-processed food (UPF) represented the proportion of calories from foods classified as UPF (based on NOVA classification) out of the total energy intake.

### Anthropometric measurements and definitions

Height and weight were measured both at baseline and at the two-year follow-up visit, from which BMI was calculated. BMI was classified according to WHO recommendations as normal weight (18.5-25 kg/m^2^), overweight (25-30 kg/m^2^), or obese (≥30 25-30 kg/m^2^). A small subset of 13 women with a BMI less than 18.5 kg/m^2^ were grouped into the normal BMI category. Given their near-normal BMI and healthy status at baseline, we believe this minor deviation does not signify actual malnutrition. For the purpose of this study, no weight change was defined as less than a 2% change at the 2-year follow-up visit. Weight loss and weight gain were defined as a 5% or greater weight reduction or gain at the two-year follow-up, respectively.

### Other measurements

Monthly household income, highest level of education, back pain for more than 3 months, moderate physical activity for at least 3 days a week, current smoking status, and recent depressive symptoms were self-reported as part of a pre-baseline visit online questionnaire. The recent depressive symptoms score (RDS) was calculated by summing four items (each scored on a 1–4 scale, where 1 = not at all, and 4 = nearly every day) assessing the presence of the following self-reported depressive symptoms over the past 2 weeks: depressed mood, unenthusiasm/disinterest, tenseness/restlessness, and tiredness/lethargy. The resulting sum score ranged between 4 and 16, with higher scores indicating more frequent and severe depressive symptoms. The RDS score has been previously validated against several commonly used depression scales, including the 9-item Patient Health Questionnaire [23].

Blood pressure was obtained at each visit while the participants were in a seated position after 5 minutes of rest. Laboratory results, including low-density lipoprotein (LDL) cholesterol, high-density lipoprotein cholesterol, total cholesterol, triglycerides, glucose, hemoglobin A1C, hemoglobin, albumin, and creatinine, were provided by participants prior to the baseline visit.

### Statistical analysis

Statistical analyses were performed using IBM SPSS Statistics for Windows, Version 29.0 (IBM Corp., Armonk, NY) and the Python programming language, Version 3.7 (Python Software Foundation, Wilmington, DE). We compared dietary habits between groups at baseline and changes at the two-year follow-up. Changes in diet at follow-up were assessed as the difference between daily consumption during the two-year follow-up and baseline. We employed the chi-squared test for binary variables and the Kruskal‒Wallis rank sum test for continuous variables. The Mann‒Whitney test was used for post hoc pairwise comparisons between the different groups. We constructed linear models by sex, to predict BMI at baseline incorporating food categories and popular foods as variables (48 food items that accounted for the highest percentage of calories logged by the participants; Supplementary Material 1: Table S3), along with age, education, income, RDS score, physical activity, and smoking status as covariates. For weight change at the 2-year follow-up, we included baseline weight as a variable, along with food categories and popular foods, with age and income as covariates (Supplementary Material 1: Table S4). A correlation matrix between the food categories and popular foods was constructed (Supplementary Material 1: Figure S1). To assess whether our study cohort is representative of healthy adults in Israel, we conducted a comparative analysis of their dietary intake against data from the Israeli national survey, MABAT, a comprehensive dietary assessment of healthy adults in Israel [15]. We focused on the 45-65 age group to closely align with our cohort. Using the mean intake of specific dietary parameters (mean daily energy intake, the percentage of total daily energy intake derived from carbohydrates, protein, and fats and absolute values for sodium) documented in MABAT [15], we established a common intake range (mean ± 25%). We then calculated the number of participants within this range, stratified by sex.

## Results

**Fig. 1:**
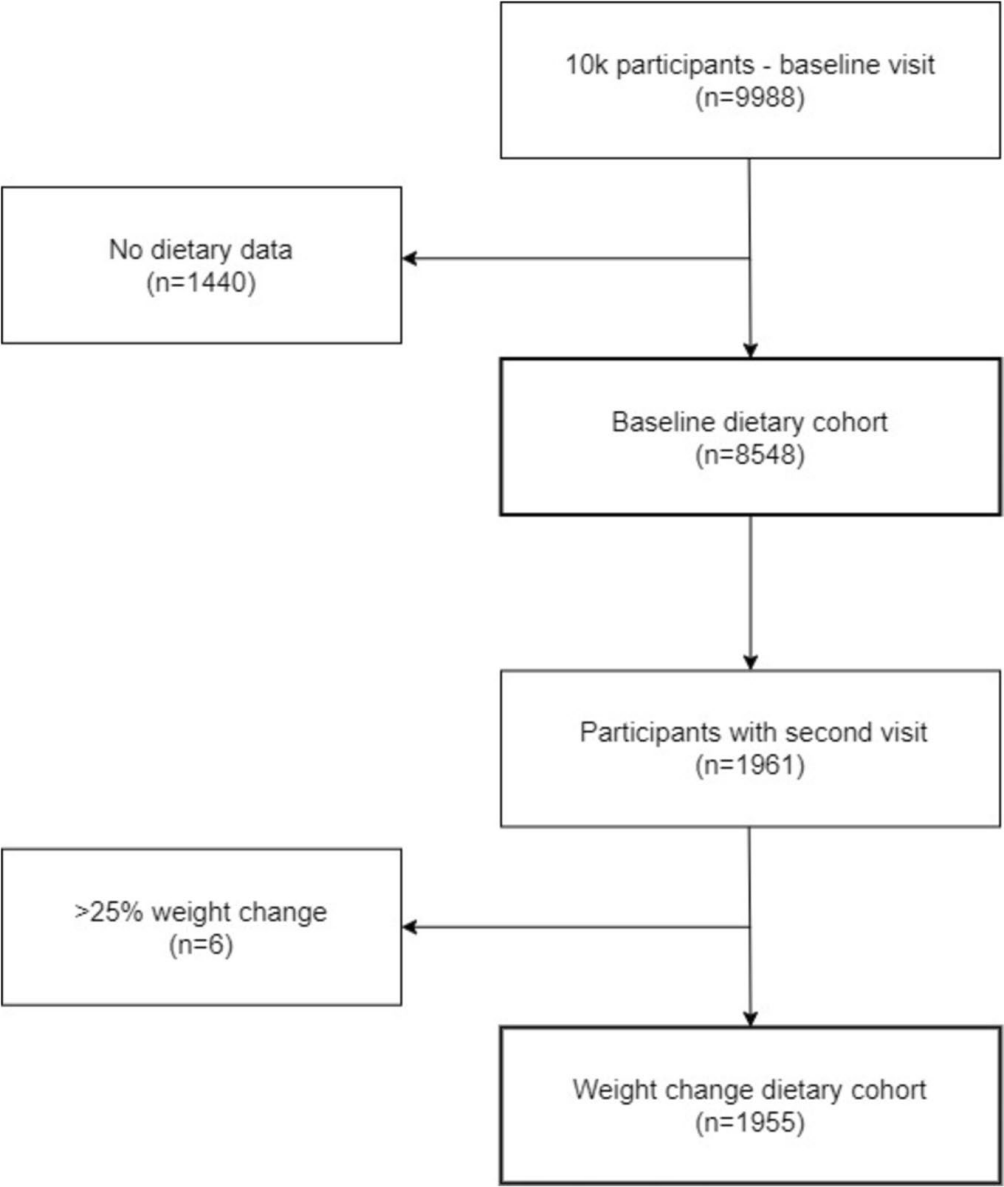
Flow diagram of the “10k cohort” participants included in this study at baseline and at the second visit

**Fig. 2:**
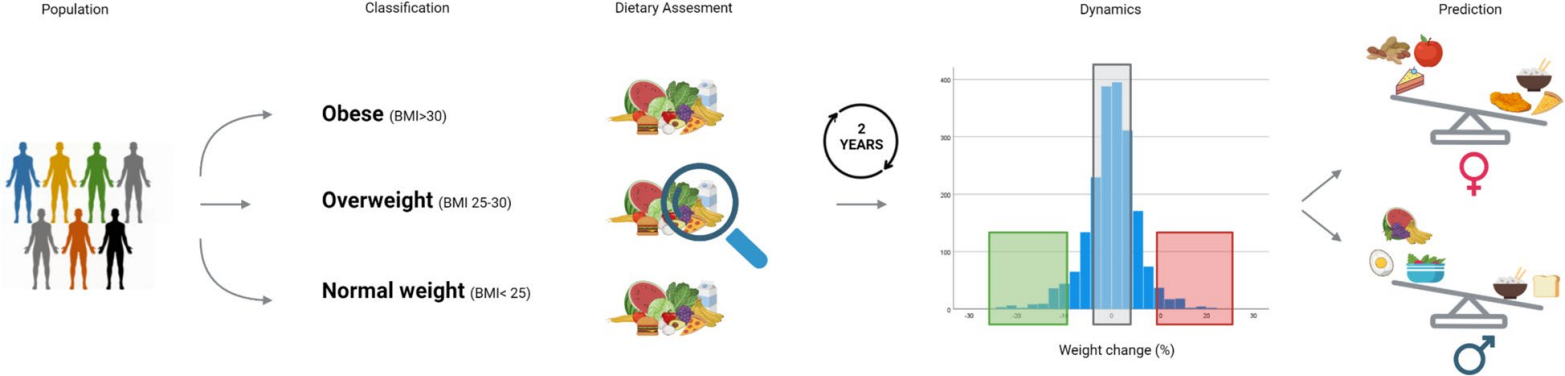
- Study description: Assessing dietary habits in different BMI groups at baseline and predicting weight change at follow-up using dietary data

Among the 9,988 individuals recruited for the 10k cohort study, 8,548 provided dietary information at the baseline visit (Figs. 1– 2). Of these, 53.1% were women. The mean age was 51.7 ± 7.8 years, and the mean BMI was 26.1 ± 4.1. Baseline demographics and clinical measurements by sex and BMI category are presented in Table 1. Among women, 34.3% were overweight and 15.5% were living with obesity, compared to 45.6% and 16.7% of men, respectively. Blood pressure and laboratory results varied across BMI categories for both sexes, with LDL cholesterol differing only among women.

**Table 1:** Anthropometric and clinical measurements by sex and BMI category at the baseline visit

	**WOMEN**				**MEN**			
	**Normal weight**	**Overweight**	**Obesity**	***p***	**Normal weight**	**Overweight**	**Obesity**	***p***
	***N*** **= 2277**	***N*** **= 1560**	***N*** **= 705**		***N*** **= 1510**	***N*** **= 1827**	***N*** **= 669**	
BMI (kg/m^2^) ± SD	22.3 ± 1.8	27.2 ± 1.4	33.2 ± 2.7		23.0 ± 1.5	27.1 ± 1.4	32.8 ± 2.4	
Age ± SD	51.2 ± 7.6	53.2 ± 7.8	53.1 ± 7.8	2.7E-16^ab^	50.3 ± 7.5	51.7 ± 7.7	52.2 ± 8.0	5.0E-10^ab^
Monthly household income				7.4E-05				0.021
6-11 k (%)	133 (5.8)	97 (6.2)	60 (8.5)		55 (3.6)	42 (2.3)	19 (2.8)	
11-15 K (%)	183 (8.0)	151 (9.7)	66 (9.4)		81 (5.4)	109 (6.1)	57 (8.5)	
15-21 k (%)	397 (17.4)	283 (18.1)	127 (18.0)		230 (15.2)	285 (15.6)	98 (14.6)	
21-36 K (%)	566 (24.9)	398 (25.4)	171 (24.3)		480 (31.8)	612 (33.5)	214 (32.0)	
> 36 K (%)	262 (11.5)	107 (6.9)	50 (7.1)		219 (14.5)	249 (13.6)	70 (10.5)	
Unknown* (%)	736 (32.3)	528 (33.8)	231 (32.8)		445 (29.5)	530 (29.0)	211 (31.5)	
Highest level of Education				1.4E-06				0.090
Unknown*(%)	47 (2.1)	25 (1.6)	13 (1.8)		33 (2.2)	39 (2.1)	22 (3.3)	
Highschool with no certificate (%)	15 (0.7)	20 (1.3)	14 (2.0)		15 (1.0)	36 (2.0)	13 (1.9)	
Matriculation certificate (%)	108 (4.7)	87 (5.6)	60 (8.5)		95 (6.3)	97 (5.3)	47 (7.0)	
Professional certificate (%)	164 (7.2)	119 (7.6)	72 (10.2)		125 (8.3)	145 (7.9)	64 (9.6)	
Bachelor’s degree (%)	726 (31.9)	467 (29.9)	210 (29.8)		529 (35.0)	674 (36.9)	237 (35.4)	
Master’s degree (%)	984 (43.2)	730 (46.8)	292 (41.5)		612 (40.5)	681 (37.3)	243 (36.3)	
PhD degree (%)	233 (10.2)	112 (7.2)	44 (6.3)		101 (6.9)	155 (8.5)	43 (6.4)	
RDS score (IQR)	2 (1–4)	2 (1–4)	3 (1–4)	0.006^bc^	2 (1–3)	2 (1–3)	2 (1–3)	0.060
Moderate physical activity at least 3 days a week. (%)	878 (47.8)	493 (40.5)	187 (34.0)	5.3E-9^abc^	653 (51.8)	669 (45.5)	181 (34.3)	1.1E-10^abc^
Current Smoking (%)	278 (14.9)	170 (13.8)	64 (11.5)	0.126	162 (12.9)	222 (15.0)	63 (11.9)	0.130
Back pain (%)	340 (14.9)	305 (19.5)	160 (22.7)	1.1E-06^ab^	293 (19.4)	375 (20.5)	152 (22.7)	0.208
Systolic BP (mmHg) ± SD	110.4 ± 14.5	116.6 ± 15.0	123.3 ± 15.5^abc^	6.2E-93^abc^	120.7 ± 13.3	127.6 ± 14.3	133.3 ± 13.8	7.7E-93^abc^
Diastolic BP (mmHg) ± SD	73.9 ± 8.9	77.9 ± 9.0	82.6 ± 9.4^abc^	6.5E-108^abc^	76.2 ± 8.9	81.1 ± 9.2	85.6 ± 9.6	6.6E-104^abc^
LDL (mg/dL) ± SD	116.7 ± 30.7	125.3 ± 30.9	128.7 ± 30.0	3.6E-14^abc^	120.7 ± 28.9	121.9 ± 28.0	120.0 ± 28.4	0.337
HDL (mg/dL) ± SD	62.5 ± 12.6	58.3 ± 12.0	54.0 ± 10.5	4.3E-51^abc^	52.4 ± 11.2	47.5 ± 10.1	43.9 ± 8.0	2.8E-64^abc^
Total cholesterol (mg/dL) ± SD	194.2 ± 35.4	203.2 ± 36.5	206.2 ± 35.4	8.4E-17^abc^	190.6 ± 36.7	192.8 ± 34.6	193.6 ± 34.6	0.037^ab^
Triglycerides (mg/dL) ± SD	85.1 ± 39.8	108.5 ± 50.1	131.6 ± 53.6	2.0E-116^abc^	100.3 ± 50.3	126.8 ± 64.6	158.2 ± 76.4	6.2E-86^abc^
Glucose (mg/dL) ± SD	90.2 ± 8.5	93.9 ± 9.7	96.7 ± 10.4	1.1E-64^abc^	92.3 ± 8.2	94.9 ± 10.0	99.4 ± 12.7	6.8E-39^abc^
HbA1C (%) ± SD	5.46 ± 1.16	5.58 ± 1.37	5.70 ± 1.47	7.0E-17^abc^	5.52 ± 1.59	5.56 ± 1.36	5.66 ± 1.34	2.8E-12^abc^
Hemoglobin (g/dL) ± SD	13.0 ± 0.9	13.2 ± 0.9	13.4 ± 0.9	3.2E-22^abc^	14.6 ± 0.9	14.8 ± 0.9	15.0 ± 0.9	4.7E-13^abc^
Albumin (g/dL) ± SD	4.23 ± 0.23	4.20 ± 0.22	4.18 ± 0.20	2.8E-06^ab^	4.38 ± 0.22	4.37 ± 0.21	4.33 ± 0.25	0.008^bc^
Creatinine (mg/dL) ± SD	0.73 ± 0.11	0.73 ± 0.10	0.73 ± 0.11	0.597	0.94 ± 0.14	0.96 ± 0.13	0.94 ± 0.15	1.4E-05^ac^

*BMI* body mass index, *BP* blood pressure, *HA1C* hemoglobin A1C, *HDL* high-density lipoprotein, *LDL* low-density lipoprotein, *RDS* recent depressive symptoms, *SD* standard deviation. Normal weight- BMI 18.5-25, Overweight- BMI 25-30, Obesity BMI≥30. Underweight -BMI<18.5 were included in the Normal weight group due to small sample size and near normal BMI

According to the post hoc analysis, there was a significant difference between the following categories: a-normal weight and overweight; b-normal weight and obese; and c-overweight and obese. * answered “don’t know”, “prefer not to say” or “less than 6,000” no data

In post hoc analysis between the different groups, there was a significant difference between a-normal weight and overweight, b-normal weight and obesity, and c-overweight and obesity

General dietary features, including energy intake and nutrients, are shown in Supplementary Material 1: Table S5. The mean daily energy intake varied by BMI category for men but not for women, decreasing from 1,831 kcal in the normal BMI category to 1,771 and 1,762 kcal in the overweight and obesity categories, respectively (*p* = 3.8E-08). Higher BMI categories in both men and women were associated with lower percentage of daily intake of carbohydrates (*p* = 0.008 for men, *p* = 2.7E-07 for women) and dietary fiber intake (*p* = 1.2E-09 for men, *p* = 8.4E-07 for women). Conversely, the percentage of daily protein intake were associated with higher BMI categories (*p* = 1.0E-24 for men, *p* = 2.3E-26 for women), while total daily fat intake did not differ significantly. Additionally, higher BMI categories were associated with increased intake of sodium, caffeine, and cholesterol.

**Fig. 3:**
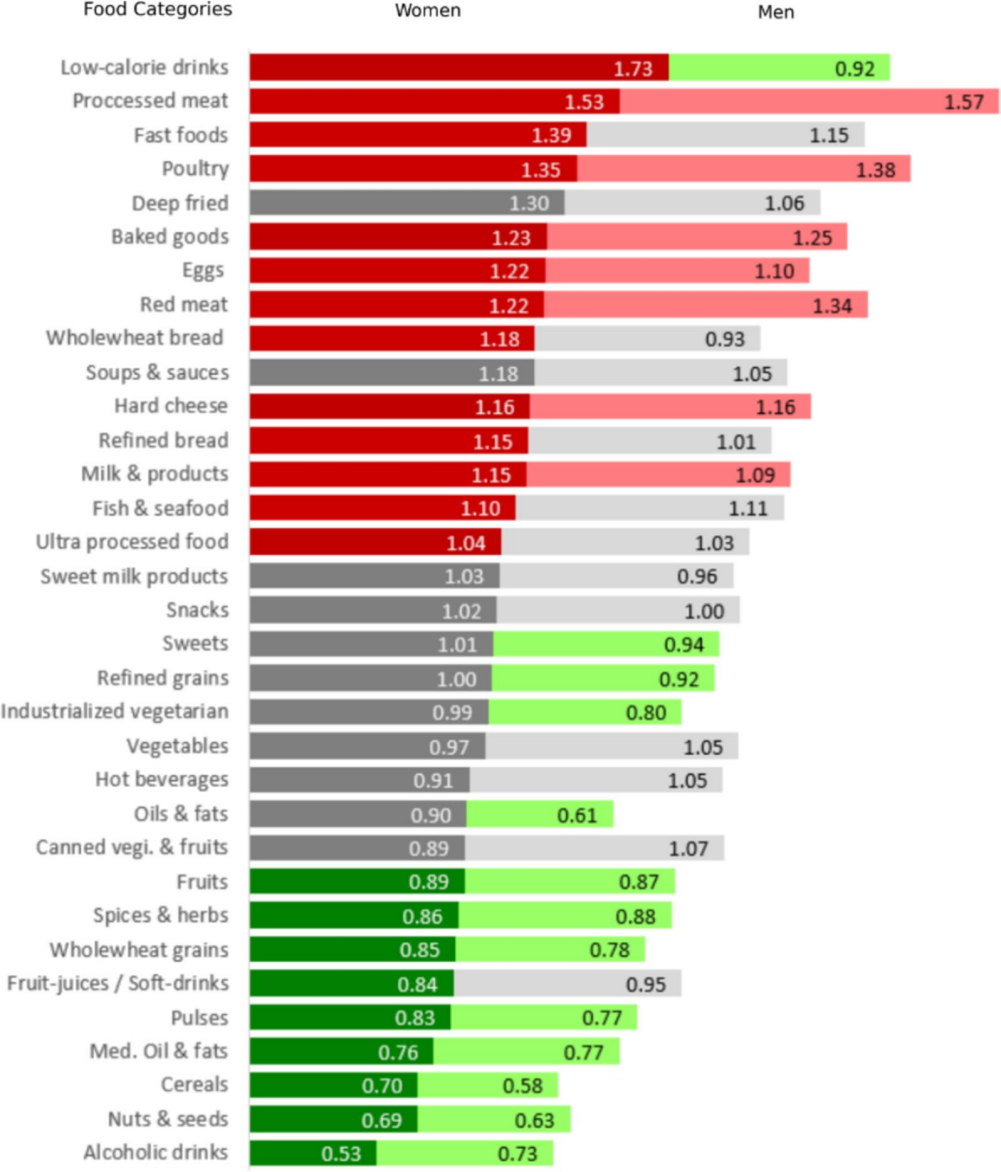
Comparison of baseline food categories consumption by BMI and sex The ratio was calculated by dividing the total daily energy consumption of each food categories between participants living with obesity and those with normal weight. Significance was determined based on comparisons shown in Table 2. Color coding: Red indicates a significant result with a Ratio > 1, representing relatively increased consumption in the obese group; Green indicates a significant result with a Ratio < 1, representing relatively decreased consumption in the obese group; and Grey indicates a nonsignificant difference. BMI- body mass index

**Fig. 4:**
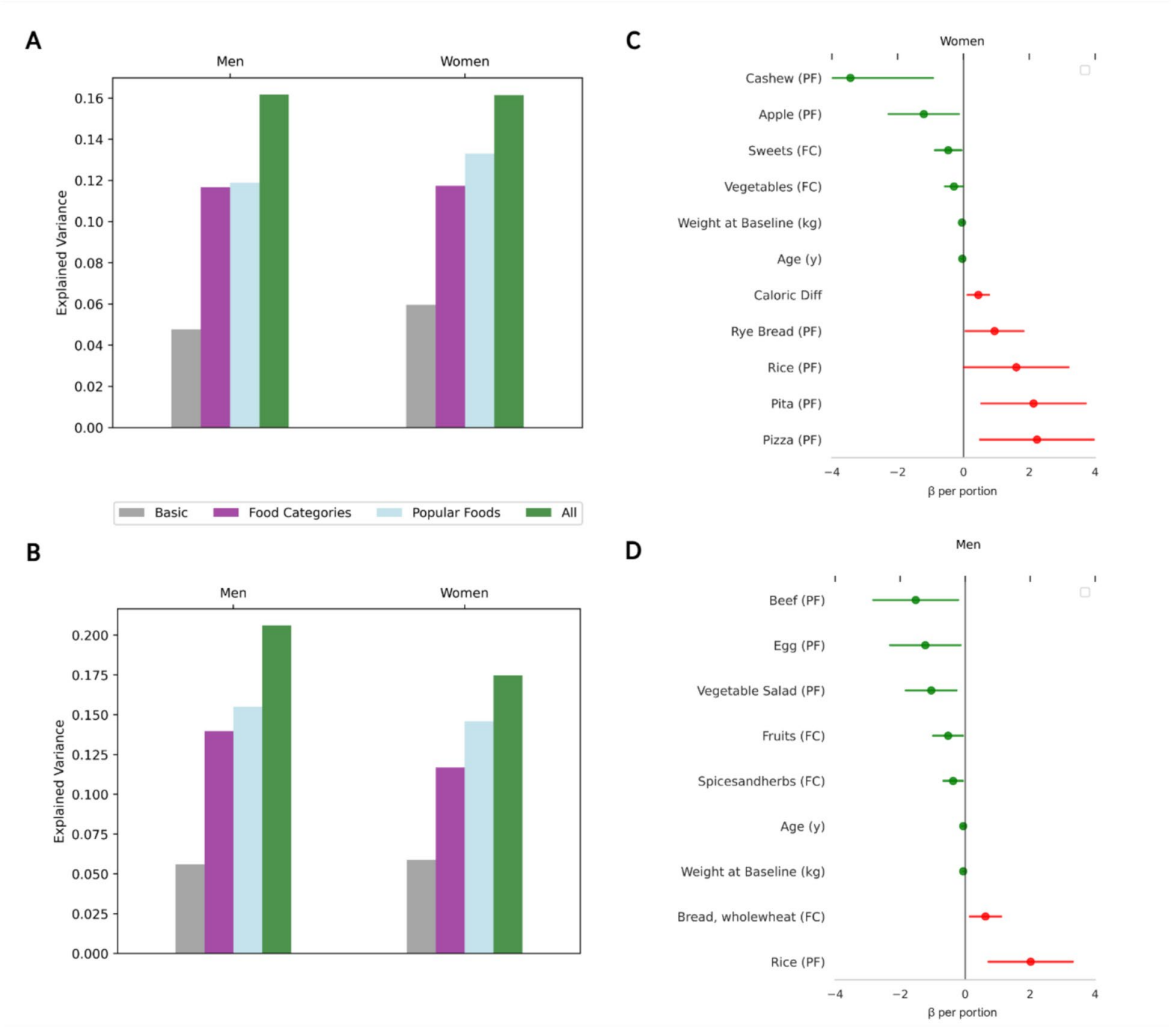
Sex-based dietary habits at baseline and prediction of weight change at follow-up **A**. Additional explained variance of food categories and popular foods at baseline **B**. and at follow-up. **C**. Regression analysis of specific foods predicting weight change for women **D**. and men. The beta coefficient is for kcal in one approximate portion. β- beta coefficient; FC- food categories; PF- popular foods

The daily intake of food categories by sex at baseline is compared in Table 2. Men and women living with obesity consumed more animal-based proteins such as eggs, milk products, and processed meat but consumed fewer plant-based proteins and fats, including pulses, nuts and seeds, and Mediterranean oils compared to their normal weight counterparts. Sex-specific habits included women living with obesity consuming nearly twice as many low-calorie drinks as did normal-weight women (173%, *p* < 0.001) and consuming 18% more calories from whole wheat bread (*p* < 0.05). Conversely, men living with obesity consumed nearly 10% fewer low-calorie drinks than did normal-weight men (*p* < 0.05) (Fig. 3). In sex-specific linear regressions for men and women, including confounders, the inclusion of food categories explained 13.6% of the BMI variance for men and 14.5% of the variance for women (Tables Supplementary Material 1: S6-S7). Similarly, the inclusion of specific foods enhanced the explained variance by 9% for men and 11% for women (Fig. 4A and Supplementary Material 1: Tables S6-S7).

**Table 2:** Daily energy intake of different food categories by sex and BMI category at baseline visit

	**WOMEN**				**MEN**			
	**Normal weight**	**Overweight**	**Obesity**	***p***	**Normal weight**	**Overweight**	**Obesity**	***p***
	***N*** **= 2277**	***N*** **= 1560**	***N*** **= 705**		***N*** **= 1510**	***N*** **= 1827**	***N*** **= 669**	
Bread- white (kcal) ± SD	156.8 ± 114.4	163.0 ± 112.9	180.7 ± 126.9	1.0E-04^abc^	228.7 ± 156.4	225.6 ± 152.6	230.3 ± 159.7	0.881
Bread- whole wheat (kcal) ± SD	43.4 ± 52.9	45.3 ± 50.4	51.2 ± 59.3	0.015^ab^	52.0 ± 71.7	44.9 ± 56.8	48.5 ± 64.4	0.705
Baked goods (kcal) ± SD	13.0 ± 26.6	13.5 ± 28.6	16.0 ± 31.6	0.042^bc^	19.0 ± 37.4	21.4 ± 39.1	23.7 ± 44.1	0.002^ab^
Cereals (kcal) ± SD	18.2 ± 43.3	14.7 ± 32.7	12.7 ± 35.7	3.1E-05^abc^	28.3 ± 58.3	24.6 ± 54.0	16.5 ± 34.2	3.5E-04^bc^
Pasta & Grains (kcal) ± SD	140.2 ± 95.6	136.7 ± 94.5	140.4 ± 89.4	0.412	182.2 ± 114.8	170.0 ± 107.3	167.9 ± 110.4	0.002^ab^
Pasta & Grains- whole wheat (kcal) ± SD	12.7 ± 26.7	10.9 ± 25.9	10.8 ± 26.8	0.003^ab^	15.2 ± 33.0	11.5 ± 28.4	11.9 ± 32.1	1.9E-04^ab^
Pulses & products (kcal) ± SD	58.9 ± 71.5	52.4 ± 61.2	49.0 ± 63.2	0.002^bc^	92.5 ± 100.7	77.4 ± 84.2	71.6 ± 83.8	6.7E-07^abc^
Fruits (kcal) ± SD	102.0 ± 90.4	96.6 ± 72.1	90.6 ± 70.5	0.041^bc^	106.3 ± 98.0	99.6 ± 90.8	92.4 ± 85.3	0.004^ab^
Vegetables (kcal) ± SD	123.9 ± 70.8	124.3 ± 69.5	120.4 ± 68.1	0.395	119.1 ± 73.9	116.5 ± 72.9	125.4 ± 84.4	0.164
Canned vegetables & fruits (kcal) ± SD	10.3 ± 18.6	9.3 ± 16.7	9.1 ± 15.5	0.717	10.5 ± 21.7	10.6 ± 23.3	11.3 ± 21.5	0.955
Industrialized vegetarian food (kcal) ± SD	5.6 ± 18.9	5.2 ± 17.2	5.5 ± 19.3	0.304	8.1 ± 26.3	5.2 ± 17.2	6.5 ± 26.0	0.001^ab^
Milk cream cheese & yogurts (kcal) ± SD	75.7 ± 69.2	82.3 ± 68.4	86.7 ± 68.8	1.2E-06^ab^	71.3 ± 79.9	73.8 ± 79.1	77.9 ± 78.4	0.014^b^
Sweet milk products (kcal) ± SD	12.3 ± 25.6	12.5 ± 26.5	12.8 ± 26.8	0.633	15.9 ± 37.8	15.0 ± 30.7	15.3 ± 30.8	0.864
Hard cheese (kcal) ± SD	42.2 ± 46.9	43.3 ± 47.1	48.9 ± 52.3	0.003^bc^	45.7 ± 59.0	46.1 ± 57.7	53.1 ± 66.4	0.037^b^
Eggs & products (kcal) ± SD	48.8 ± 40.7	53.7 ± 41.5	59.5 ± 45.4	3.5E-10^abc^	57.0 ± 52.8	57.1 ± 51.0	62.4 ± 49.3	0.001^bc^
Fish & seafood (kcal) ± SD	55.2 ± 56.6	59.2 ± 60.1	60.7 ± 60.2	0.007^ab^	62.6 ± 61.3	66.3 ± 64.6	69.4 ± 66.4	0.052
Poultry & products (kcal) ± SD	58.9 ± 71.5	52.4 ± 61.2	49.0 ± 63.2	1.4E-23^abc^	92.5 ± 100.7	77.4 ± 84.2	71.6 ± 83.8	1.9E-23^abc^
Red meat (kcal) ± SD	47.8 ± 63.5	54.5 ± 62.6	58.3 ± 67.6	5.2E-08^ab^	90.7 ± 115.6	101.1 ± 105.9	121.3 ± 117.1	6.0E-14^abc^
Processed meat (kcal) ± SD	8.9 ± 2.1	11.4 ± 25.0	13.6 ± 27.0	1.5E-11^abc^	16.9 ± 32.5	25.3 ± 45.9	26.6 ± 43.8	1.4E-13^ab^
Oils & fats (kcal) ± SD	27.1 ± 49.9	24.2 ± 39.2	24.3 ± 36.7	0.999	31.9 ± 67.8	24.3 ± 59.2	19.3 ± 40.4	6.6E-05^ab^
Mediterranean Oil & fats (kcal) ± SD	60.8 ± 70.6	47.8 ± 50.9	46.3 ± 50.7	7.6E-10^ab^	77.3 ± 91.0	63.6 ± 73.6	59.3 ± 69.8	4.3E-08^ab^
Nuts seeds & products	83.1 ± 96.6	69.1 ± 82.5	57.6 ± 64.6	1.2E-09^abc^	102.4 ± 126.8	78.5 ± 106.4	64.8 ± 88.5	4.2E-12^abc^
Hot beverages (kcal) ± SD	36.6 ± 49.9	36.8 ± 49.2	33.2 ± 41.1	0.793	30.9 ± 46.4	33.5 ± 44.3	32.4 ± 47.3	0.002^a^
Drinks – low calorie & diet (kcal) ± SD	0.25 ± 1.87	0.36 ± 3.30	0.44 ± 2.24	0.002^ab^	0.42 ± 5.14	0.56 ± 4.55	0.39 ± 2.24	0.013^ab^
Drinks – Fruit juices & soft drinks (kcal) ± SD	14.0 ± 28.4	12.4 ± 24.5	11.7 ± 25.0	0.041^b^	23.1 ± 40.6	21.7 ± 39.2	21.9 ± 60.8	0.366
Drinks – Alcohol (kcal) ± SD	26.4 ± 43.7	21.3 ± 39.9	13.9 ± 32.7	1.4E-17^abc^	45.9 ± 61.8	48.5 ± 67.7	33.5 ± 52.8	2.5E-06^bc^
Deep fried foods (kcal) ± SD	4.6 ± 13.7	5.6 ± 18.8	6.0 ± 17.5	0.638	7.1 ± 17.3	7.9 ± 19.8	7.5 ± 21.0	0.585
Fast foods (kcal) ± SD	8.0 ± 19.1	9.9 ± 25.3	11.1 ± 25.5	0.006^ab^	19.9 ± 36.4	22.0 ± 40.9	22.9 ± 39.3	0.148
Snacks (kcal) ± SD	16.8 ± 31.8	17.4 ± 33.5	17.2 ± 34.3	0.983	18.7 ± 40.3	18.4 ± 32.7	18.7 ± 36.0	0.298
Sweets (kcal) ± SD	200.5 ± 135.0	201.8 ± 146.3	201.6 ± 141.8	0.746	236.0 ± 164.4	237.1 ± 169.0	221.6 ± 171.1	0.020^bc^
Soups & sauces (kcal) ± SD	10.0 ± 15.3	11.1 ± 17.2	11.8 ± 17.4	0.157	11.2 ± 27.2	10.0 ± 18.2	11.7 ± 19.7	0.308
Spices & herbs (kcal) ± SD	1.02 ± 3.66	0.86 ± 3.29	0.88 ± 3.81	0.007^ab^	1.32 ± 4.71	0.90 ± 3.51	1.16 ± 9.59	4.4E-07^ab^
Ultra processed foods (%) ± SD	24.2 ± 11.1	24.8 ± 10.7	25.2 ± 10.5	0.012^ab^	24.9 ± 10.9	25.6 ± 10.7	25.6 ± 11.3	0.079
Other	3.21 ± 9.94	2.41 ± 8.81	2.08 ± 8.84	1.2E-04^ab^	3.79 ± 13.39	1.97 ± 8.10	1.51 ± 5.55	1.2E-06^ab^

*BMI* body mass index, *SD* standard deviation. Normal weight- BMI 18.5-25, Overweight- BMI 25-30, Obesity BMI≥30. Underweight -BMI<18.5 were included in the Normal weight group due to small sample size and near normal BMI

Post hoc analysis revealed a significant difference between the a-normal weight and b-normal weight groups and between the c-overweight and obesity groups

We compared macronutrient, micronutrient, and food categories among participants who lost weight, gained weight, or had no weight change at follow-up (Tables 3, and Supplementary Material 1: Tables S8-S10). On average, participants logged fewer daily calories at the two-year follow-up than at baseline. Women who lost weight decreased their daily energy intake the most (-206 kcal) compared to those with no change (-160 kcal) and weight gain (-115 kcal) (*p* = 0.012). In contrast, compared with men with no weight change, men who lost weight did not differ in their caloric deficit but did significantly reduce their daily carbohydrate consumption (-4.1 ± 9.2 vs. -1.3 ± 6.4, *p* < 0.05). The daily percentage of protein and fat intake did not differ for either women or men.

**Table 3:** Changes in daily energy intake of different food categories by sex and weight change category

	**WOMEN**				**MEN**			
	**No weight change**	**Weight loss**	**Weight gain**	***p***	**No weight change**	**Weight loss**	**Weight gain**	***P***
	***N*** ** = 338**	***N*** ** = 162**	***N*** ** = 157**		***N*** ** = 384**	***N*** ** = 104**	***N*** ** = 111**	
Bread- white (kcal) ± SD	-7 ± 110.8	-34.1 ± 134.4	5.7 ± 114.9	0.107	-16.2 ± 142.6	-35.6 ± 136.5	31.2 ± 170.5	0.035^c^
Bread- whole wheat (kcal) ± SD	-9 ± 54.7	-7.8 ± 64.6	-10 ± 53.7	0.987	-7.5 ± 59.7	-22 ± 60.2	-5.6 ± 70.6	0.097
Baked goods (kcal) ± SD	2.1 ± 33.6	-2.5 ± 32.6	2.1 ± 27.7	0.366	1.8 ± 51.8	-4.9 ± 36	7.1 ± 50.8	0.037^ac^
Cereals (kcal) ± SD	0.1 ± 37.4	-6.5 ± 24.8	2.6 ± 33.1	0.015^ac^	-6.9 ± 50.6	-12 ± 57.1	-9 ± 47.2	0.886
Pasta & Grains (kcal) ± SD	-14.5 ± 87.4	-40.1 ± 93.7	-7.7 ± 119.6	0.012^ac^	-28.9 ± 117.7	-62.5 ± 108.9	-3.4 ± 119.8	0.001^ac^
Pasta & Grains- whole wheat (kcal) ± SD	-0.6 ± 33.4	0.6 ± 32.8	-5.2 ± 36.2	0.369	-4.9 ± 25.3	0.2 ± 57	-1.7 ± 32.4	0.108
Pulses & products (kcal) ± SD	-6.2 ± 61.9	-12.7 ± 69.3	-22.5 ± 71.9	0.176	-21.2 ± 84.9	-2.1 ± 103.4	-14.2 ± 93.9	0.111
Fruits (kcal) ± SD	-22.4 ± 54	-27.2 ± 65.3	-23 ± 62.1	0.688	-30 ± 72.5	-3.2 ± 99.2	-50.3 ± 82.6	0.020^c^
Vegetables (kcal) ± SD	-12.6 ± 69.9	-6 ± 64.2	-10 ± 61.4	0.606	-11.8 ± 70.2	14.8 ± 70.2	-10.7 ± 81.9	0.001^ac^
Canned vegetables & fruits (kcal) ± SD	-3.6 ± 21.2	-0.5 ± 29.3	-4.4 ± 20	0.825	-5.3 ± 22	-4.8 ± 19.3	-2.3 ± 19.6	0.663
Industrialized vegetarian food (kcal) ± SD	-1.9 ± 20.7	0.5 ± 16.4	0.8 ± 16.7	0.5	-0.6 ± 19.8	1.8 ± 25.9	1.6 ± 14.5	0.441
Milk cream cheese & yogurts (kcal) ± SD	-15.9 ± 65.3	-19.5 ± 61.9	-6.9 ± 63.4	0.068	-15.1 ± 71	-12.7 ± 73.3	-12.5 ± 75.2	0.615
Sweet milk products (kcal) ± SD	-1.7 ± 29.1	-2.8 ± 27.7	0.5 ± 30.2	0.32	-1.7 ± 32.6	-5.2 ± 30.7	0.7 ± 28.7	0.202
Hard cheese (kcal) ± SD	-3.6 ± 43	-3.7 ± 52.1	-2 ± 59.3	0.668	-2.4 ± 58	10.8 ± 86.8	-9.2 ± 92.7	0.537
Eggs & products (kcal) ± SD	-0.4 ± 50.6	-0.3 ± 51.5	-0.4 ± 48	0.573	-5.1 ± 45.2	5.6 ± 50	6.2 ± 61.2	0.084
Fish & seafood (kcal) ± SD	-7.6 ± 57.5	-4.9 ± 61.5	2.4 ± 64.5	0.371	-8.6 ± 63.1	-6.1 ± 73.6	-14 ± 75.7	0.872
Poultry & products (kcal) ± SD	-1 ± 75.9	-13.7 ± 73.5	3.8 ± 75.7	0.027^ac^	-4.9 ± 98.4	-16.5 ± 96.1	-16.8 ± 106.6	0.219
Red meat (kcal) ± SD	-6.8 ± 67.8	5.8 ± 75.4	-2.2 ± 59.9	0.083	-10.7 ± 89.9	-2.4 ± 102	-32 ± 165.6	0.114
Processed meat (kcal) ± SD	-2.3 ± 24.3	-2.1 ± 26.6	-3.9 ± 32.9	0.968	0.6 ± 40.9	-6 ± 60.8	3.7 ± 76.9	0.282
Oils & fats (kcal) ± SD	-5.1 ± 37.4	-6.5 ± 46.6	-11.2 ± 41	0.414	-7.6 ± 42.9	-7.1 ± 77.4	-27 ± 68.2	0.108
Mediterranean Oil & fats (kcal) ± SD	-7.3 ± 54.1	4 ± 63.3	-9 ± 54.5	0.407	-0.1 ± 72.6	2.6 ± 70.3	-21.1 ± 77.4	0.027^bc^
Nuts seeds & products	-3.6 ± 74.9	-18.9 ± 113	-20.6 ± 91.6	0.04^b^	-23.3 ± 105.3	22.9 ± 151.3	-37.9 ± 130.9	0.003^ac^
Hot beverages (kcal) ± SD	-4.1 ± 43.7	0.6 ± 46.2	-0.3 ± 38.9	0.548	0.3 ± 40.4	-3 ± 42.7	-3.5 ± 42.3	0.574
Drinks – low calorie & diet (kcal) ± SD	0.1 ± 4.1	-0.5 ± 4.2	0 ± 1.1	0.141	-0.3 ± 2.3	-0.6 ± 3.9	-0.4 ± 3	0.985
Drinks – Fruit juices & soft drinks (kcal) ± SD	-3.1 ± 27.4	-4.3 ± 27.3	-2.5 ± 27.1	0.783	-5 ± 32.1	-13.9 ± 29.8	-11.9 ± 43.4	0.037^a^
Drinks – Alcohol (kcal) ± SD	-8.2 ± 34.6	0.7 ± 29.2	-3.9 ± 24.6	0.061	-11.9 ± 43.4	-11.2 ± 33.2	-8.3 ± 39	0.327
Deep fried foods (kcal) ± SD	-1.2 ± 19.6	-3.5 ± 27.9	-2.9 ± 18.6	0.753	-0.4 ± 28.6	-1.8 ± 22.3	-0.5 ± 25.3	0.756
Fast foods (kcal) ± SD	0.6 ± 27.6	-5.5 ± 26.4	0.4 ± 21	0.768	-1.2 ± 47.8	-11.6 ± 55.4	-2.3 ± 44.6	0.474
Snacks (kcal) ± SD	-4.4 ± 34	-1.9 ± 34.7	9.5 ± 57.5	0.047^b^	-1.4 ± 36	0.3 ± 38	2 ± 35.9	0.772
Sweets (kcal) ± SD	-39.1 ± 132.6	-31.8 ± 145.6	-31.3 ± 173.1	.3600	-58.5 ± 154.5	-53.6 ± 134.9	-57.5 ± 146.5	0.765
Soups & sauces (kcal) ± SD	-3.9 ± 17.3	-2.6 ± 18.7	-1.6 ± 18.8	0.647	-2.4 ± 19	-2.9 ± 19.9	-2.7 ± 20.9	0.654
Spices & herbs (kcal) ± SD	-0.9 ± 6.7	-0.6 ± 5.7	-0.6 ± 6.3	0.508	-0.6 ± 4.8	1.3 ± 9.2	-0.8 ± 3.2	0.204
Ultra processed foods (%) ± SD	0.07 ± 10.7	0.68 ± 11.8	1.8 ± 12.2	0.471	0 ± 10.3	-3.2 ± 10.9 ^(n=103)^	1.8 ± 11.4	0.002^ac^
Other	-0.3 ± 7.2	-0.2 ± 8.6	0.4 ± 12.9	0.449	-1.2 ± 9.3	-0.7 ± 11.5	-1.1 ± 10.5	0.545

*SD* standard deviation. No weight change was defined as a < 2% change at follow-up. Weight loss ≥ 5% weight reduction at follow-up. Weight gain ≥ 5% weight gain at follow-up. Post hoc analysis revealed a significant difference between a- no change and weight loss, b- no change and weight gain, and c- weight loss and weight gain

Compared with those who gained more than 5%, men who lost more than 5% of their initial weight decreased energy intake from refined bread products and UPFs (-35.6 ± 136.5 vs. 31.2 ± 170.5; *p* > 0.05 for bread, -3.2 ± 10.9 vs. 1.8 ± 11.4; *p* < 0.05 for UPFs). Women who lost weight decreased their poultry and cereal consumption compared to those who gained weight (Table 3).

In a linear regression analysis predicting percent weight change, adjusted for age, education, income, RDS score, physical activity, and smoking status, these factors explained only 5.6% of the variance for men and 5.8% for women. Adding food categories and specific foods increased the explained variance to 20.6% for men and 17.5% for women (Fig. 4B). For men, adding 1 cup of rice (~ 220 kcal) daily was linked to a 2.5% weight gain, and 1 slice of whole wheat bread slightly increased weight. Conversely, 1 egg (~ 80 kcal) was linked to a 1.2% weight decrease, and 1 portion of beef (~ 265 kcal) was linked to a 1.5% decrease, with fruits and vegetable salad having a small effect. In women, 1 pita bread (~ 200 kcal) was associated with a 2.1% weight gain, 1 slice of pizza (~ 300 kcal) with a 2.2% gain, and 1 portion of rice with a 1.6% gain, as well as rye bread. Conversely, 1 apple (~ 80 kcal) was linked to a 1.2% weight decrease, and a handful of cashew nuts (~ 250 kcal) were linked to a 3.4% decrease, with vegetables having a slight effect. Interestingly, changes in total energy intake contributed to the model in women but not men (Figs. 4C-4D**, **and Supplementary Material 1: Tables S11-S12).

## Discussion

In this large observational prospective study, we explored the dietary habits of 8,548 healthy participants from the 10K Project and their potential effects on baseline BMI and weight change at two years of follow-up. The analysis revealed sex-specific dietary habits among men and women across various BMI categories. Interestingly, women, regardless of BMI, reported similar energy consumption. In contrast, men with a normal BMI consumed approximately 4% more calories daily than those living with overweight or obesity. Additionally, among women, caloric differences were linked to two-year weight changes, a trend not observed in men. These findings suggest that factors beyond energy intake alone are crucial for predicting weight status and highlight the complexity of weight management.

While the literature extensively documents differences in dietary intake between sexes, and some government dietary recommendations reflect these variations [24], there remains a notable lack of sex-specific weight loss guidelines. Previous research has shown sex-based dietary preferences and behaviors. For example, the UK Biobank, a large-scale cohort reported sex differences in adherence to dietary recommendation including specific nutrients that were more likely to be consumed by men or women [25]. In our research, we observed sex-based dietary habits both at baseline and at the two-year follow-up. Notably, separate models for each sex more effectively explained the variance in these changes compared to a single model that treated sex as an independent covariate, even after controlling for confounders (Fig. 4A-4B). In our cohort, fruits and vegetables were generally associated with reduced weight, while rice and various breads were associated with increased weight. Men seemed to benefit from increasing their consumption of animal-based protein, while women seemed to benefit more from plant-based fat (Fig. 4C-4D). These findings align with studies reporting health benefits for men increasing animal-based protein [26]. Interestingly, it has been reported that women have a stronger belief that fruits are an important factor in health compared to men [27]. In addition, men may hold more favorable views towards incorporating animal-based proteins into their diets, whereas women are generally more inclined to adopt plant-based diets [28]. These insights can guide individuals in making more informed choices when pursuing a healthier lifestyle.

We chose to convey the results in change per portion since we believe it can help make our data more accessible. For example, for a woman, an apple and a handful of cashews a day can help achieve up to a 5% weight loss. Interestingly, in this current study an increase in sweets consumption has been linked to weight loss in women. Although this finding may seem counterintuitive, certain sweets, such as chocolate, have been reported to offer health benefits and contribute to weight reduction [18, 29, 30].

We utilized a smartphone application for advanced digital data collection of continuous dietary information. This approach offers a more accurate assessment of participants' actual dietary habits, overcoming the limitations of traditional dietary intake assessment tools, such as food frequency questionnaires and occasional 24-h recalls [31, 32]. Moreover, we were able to report specific foods and food categories along with macronutrients to provide a comprehensive assessment. However, the inherent challenges of self-reported dietary data remain, such as potential avoidance of certain foods during the logging period or underreporting of specific categories. As reported in previous studies, food logging in a digital app might still end up with underestimation of food and energy consumption [33]. In our study, for example, we observed a lower intake of sweets among men with obesity compared to men of normal weight. This finding may partly reflect underreporting of 'unhealthy' foods by individuals living with obesity. Nonetheless, digital applications provide the advantage of, easy, continuous, real-time food logging, which may improve the availability and accuracy of dietary data [34, 35].

Our observational cohort had a greater proportion of overweight men (45%) than women (30.3%), potentially skewing outcomes towards a less healthy profile for males. Yet, these differences reflect real-world dynamics within the Israeli population and align with the national MABAT survey of healthy adults in Israel [15], particularly for men (Supplementary Material 1: Table S13). Additional research is needed to generalize our results and explore the underlying reasons for these sex disparities.

Other limitations include the reliance on BMI to define obesity, which, while cost-effective and associated with various lifestyle factors and clinical outcomes [4], does not account for body composition, a critical health determinant [36–38]. Future investigations should include body composition for a more comprehensive understanding. Finally, our study focused on healthy individuals aged 40–70 years, so the findings may not extend to other demographic groups, warranting further exploration.

## Conclusions

This large prospective study revealed notable sex-based differences in dietary habits and their effects on weight change over time. These findings suggest that dietary interventions should consider these differences to enhance weight management strategies. Future trials are needed to further investigate the emerging patterns from these habits and their implications for personalized nutrition and health outcomes.

### Supplementary Information

Supplementary Material 1: Tables S1-13, Fig. S1. Tables S1-13 and Figure S1. Table S1: A list of Food Categories. Table S2: Items included in the “Other” Food category. Table S3: List of popular Foods. Table S4: List of variables in regression analysis. Table S5: Daily nutrient intake by gender and BMI categories at baseline visit. Table S6: Regression analyses predicting baseline BMI in women. Table S7: Regression analyses predicting baseline BMI in men. Table S8: Anthropometric and clinical measurements by gender and weight difference at two-year follow-up. Table S9: Comparing first visit and follow-up cohort demographics. Table S10: Daily nutrient intake by gender and weight and difference at two-year follow-up. Table S11: Regression analyses predicting weight change in women at follow-up. Table S12: Regression analyses predicting weight change in men at follow-up. Table S13: Percentage of '10K Cohort' participants within Israeli ‘Mabat Survey’ ranges. Figure S1: Correlation of Food categories and popular foods.

## Supplementary Information

Supplementary Material 1: Tables S1-13, Fig. S1.  Tables S1-13 and Figure S1. Table S1: A list of Food Categories. Table S2: Items included in the“Other” Food category. Table S3: List of popular Foods. Table S4: List of variables in regression analysis. Table S5: Daily nutrient intake by gender and BMI categories at baseline visit. Table S6: Regression analyses predicting baseline BMI in women. Table S7: Regression analyses predicting baseline BMI in men. Table S8: Anthropometric and clinical measurements by gender and weight difference at two-year follow-up. Table S9: Comparing first visit and follow-up cohort demographics. Table S10: Daily nutrient intake by gender and weight and difference at two-year follow-up. Table S11: Regression analyses predicting weight change in women at follow-up. Table S12: Regression analyses predicting weight change in men at follow-up. Table S13: Percentage of '10K Cohort' participants within Israeli ‘Mabat Survey’ ranges. Figure S1: Correlation of Food categories and popular foods.

## Data Availability

The data are accessible to researchers from universities and other research institutions at https://humanphenotypeproject.org/home.

## Abbreviations

BMI: Body mass index
LDL: Low-density lipoprotein
MUFAs: Monounsaturated fatty acids
NIH: National Institutes of Health
PUFAs: Polyunsaturated fatty acids
SatFat: Saturated fatty acids
UPF: Ultra-processed food
