# Supplementary material for: Sex-specific dietary habits and their association with weight change in healthy adults
Source: BMC Med. 2024 Nov 6;22:512. doi: 10.1186/s12916-024-03730-3 (PMC11539530; doi:10.1186/s12916-024-03730-3)
Supplement: Supplementary file 1 — Supplementary Material 1: Tables S1-13, Fig. S1. Tables S1-13 and Figure S1. Table S1: A list of Food Categories. Table S2: Items included in the“Other” Food category. Table S3: List of popular Foods. Table S4: List of variables in regression analysis. Table S5: Daily nutrient intake by gender and BMI categories at baseline visit. Table S6: Regression analyses predicting baseline BMI in women. Table S7: Regression analyses predicting baseline BMI in men. Table S8: Anthropometric and clinical measurements by gender and weight difference at two-year follow-up. Table S9: Comparing first visit and follow-up cohort demographics. Table S10: Daily nutrient intake by gender and weight and difference at two-year follow-up. Table S11: Regression analyses predicting weight change in women at follow-up. Table S12: Regression analyses predicting weight change in men at follow-up. Table S13: Percentage of '10K Cohort' participants within Israeli ‘Mabat Survey’ ranges. Figure S1: Correlation of Food categories and popular foods. [file 12916_2024_3730_MOESM1_ESM.docx]

**Supplementary Material 1**

**Title: Gender-Specific Dietary Patterns and Their Association with Weight Change in Healthy Adults.**

Authors: Michal Rein, Matan Elkan, Nastya Godneva, Noa Dolev, Eran Segal.

Table of contents:

Table S1…………………………………....…………………………………….................……......................…………2

Table S2………....……………………………………………….................…………………….……….......................3

Table S3…………………………………………………………....……...…................…………….......................…..6

Table S4…………………………………………………………....……...…................…………….......................…..7

Table S5……………………………………………………………....……...…...............……..….....................…......9

Table S6……………………………………………………………....……...…...............……..…...................…......10

Table S7……………………………………………………………....……...…...............……..….....................…....11

Table S8……………………………………………………………....……...…...............……..….....................…....12

Table S9……………………………………………………………....……...…...............……..….....................…....12

Table S10…………………………………………………………....……...…...............……..….....................…....13

Table S11…………………………………………………………....……...…...............……..….....................….....15

Table S12…………………………………………………………....……...…...............……..….....................….....15

Table S13…………………………………………………………....……...…...............……..….....................….....16

Figure S1…………………………………………………………....……...…...............……..….....................….....17

**Table S1: A list of Food Categories from 10k cohort dietary app.**

| 1 | Hard cheese |
| --- | --- |
| 2 | Milk, cream cheese and yogurts |
| 3 | Sweet milk products |
| 4 | Beef, veal, lamb, and other meat products (Red meat) |
| 5 | Poultry and its products |
| 6 | Fish and seafood |
| 7 | Processed meat products |
| 8 | Nuts, seeds, and products |
| 9 | Eggs and their products |
| 10 | Cereals |
| 11 | Cereals wholewheat |
| 12 | Sweets |
| 13 | Baked goods |
| 14 | Snacks |
| 15 | Pasta, Grains and Side dishes |
| 16 | Pasta, Grains and Side dishes wholewheat |
| 17 | Pulses and products |
| 18 | Bread |
| 19 | Bread wholewheat |
| 20 | Industrialized vegetarian food |
| 21 | Soups and sauces |
| 22 | Fruits |
| 23 | Vegetables |
| 24 | Canned vegetables and fruits |
| 25 | Oils and fats |
| 26 | Mediterranean Oil and fats |
| 27 | Hot beverages |
| 28 | Alcoholic Drinks |
| 29 | Fruit juices and soft drinks |
| 30 | Low calories and diet drinks |
| 31 | Fast Foods |
| 32 | Spices and herbs |
| 33 | Ultra processed foods |
| 34 | Others |

**Table S2: Items included in the “Other” Food category.**

| **English Name** | | | | |
| --- | --- | --- | --- | --- |
| Compressed fresh yeast |  |  |  |  |
| Cocoa powder without dried milk |  |  |  |  |
| Powdered lemon pudding |  |  |  |  |
| Saccharomyces sweetener tablets |  |  |  |  |
| Saccharin-based sugar substitute Diatype liquid |  |  |  |  |
| Bee pollen |  |  |  |  |
| half sugar sucrose |  |  |  |  |
| Unsweetened dry powder pectin |  |  |  |  |
| Artificial vanilla extract |  |  |  |  |
| Maca powder |  |  |  |  |
| Thelma pollen orchid |  |  |  |  |
| A sugar substitute based on stevia powder |  |  |  |  |
| cocoa mass |  |  |  |  |
| Aspartame sugar substitute powder |  |  |  |  |
| Maltitol |  |  |  |  |
| Instant pudding vanilla powder |  |  |  |  |
| Cocoa powder 10% fat |  |  |  |  |
| Classic vegan cappuccino powder |  |  |  |  |
| Milk-free powdered coffee whitener including CREMORA.COFFEEMATE | | |  |  |
| powdered lemonade |  |  |  |  |
| Powder for making carob drink, carob organic shake B&D | | | | |
| Lecithin |  |  |  |  |
| Chrolophyll |  |  |  |  |
| Cocoa beans |  |  |  |  |
| coconut flour |  |  |  |  |
| Gluten-free Mix it flour Shar |  |  |  |  |
| Tea is prepared from sweetened instant powder before sweetener | | |  |  |
| B&D carob powder |  |  |  |  |
| nescafe dolce gusto |  |  |  |  |
| Carob powder |  |  |  |  |
| Brewer |  |  |  |  |
| Haaretz flour Molino, multi-purpose mixes without molino | | | | |
| Dry yeast |  |  |  |  |
| Calcium lactate CALCIUM LACTATE |  |  |  |  |
| Elite Milk Coffee Whitener |  |  |  |  |
| Organic raw cocoa powder of grains |  |  |  |  |
| Sugar substitute fructose sweetener powder |  |  |  |  |
| Psyllium husks |  |  |  |  |
| Vinegar |  |  |  |  |
| Elite low-calorie cocoa |  |  |  |  |
| An orchid is prepared from a water-based powder |  |  |  |  |
| Collagen powder |  |  |  |  |
| TUSSO vanilla flavored instant pudding |  |  |  |  |
| Cream of tartar |  |  |  |  |
| Dashi powder |  |  |  |  |
| Lemon peel |  |  |  |  |
| Spirulina powder |  |  |  |  |
| Liquid sucralose sugar substitute |  |  |  |  |
| Baking soda powder |  |  |  |  |
| GFREE instant vanilla pudding |  |  |  |  |
| Sugar substitute based on liquid stevia |  |  |  |  |
| Pudding for cooking vanilla or strawberry flavored powder | | | | |
| Sweet and light sugar substitute SWEET 'N LOW saccharin powder/tablets | | | |  |
| Ground cocoa beans 100% cocoa almond Tabor |  |  |  |  |
| Gelatin dry powder |  |  |  |  |
| SPLENDA sucralose sugar substitute including bags |  |  |  |  |
| Stevia sweetener |  |  |  |  |
| Brown sugar substitute |  |  |  |  |
| Gold Sucrose Sweetener Tablets |  |  |  |  |
| Baking powder |  |  |  |  |
| Sugar substitute based on stevia tablets |  |  |  |  |
| Liquid saccharin base sugar substitute |  |  |  |  |
| Advanced protein combination protein 3D |  |  |  |  |
| Coconut milk powder |  |  |  |  |
| Sugar substitute based on powdered saccharin |  |  |  |  |
| Sucralose-based sugar substitute Sucralite tablets |  |  |  |  |
| Fat-free milk powder without added vitamin A and vitamin D | | | | |
| Salt |  |  |  |  |
| GUAR GUM |  |  |  |  |
| Cocoa powder 24% fat |  |  |  |  |
| Maki berry powder |  |  |  |  |
| A saccharin-based sugar substitute such as powdered sucrose | | |  |  |
| Saccharin-based sugar substitute for the Ramshi tablets | | | | |
| Toffee vegan cappuccino powder |  |  |  |  |
| Low calorie powdered sugar substitute |  |  |  |  |
| Chewing gum without coating without sugar |  |  |  |  |
| diabetes diet |  |  |  |  |
| Brown sugar |  |  |  |  |
| Moringa,willow moringa leaf powder | | | | |
| Orbit White gum without sugar with fruit flavor |  |  |  |  |
| Frozen spirulina |  |  |  |  |
| Mentos gum without sugar, fine mint | | | | |
| Cocoa chips |  |  |  |  |
| Isopure protein powder |  |  |  |  |
| Omega 3 |  |  |  |  |
| Turmeric extract and black pepper |  |  |  |  |
| Preworkout caffeine supplement |  |  |  |  |
| Vitamin C 500 |  |  |  |  |
| Vitamin D3 |  |  |  |  |
| B-complex 50 |  |  |  |  |
| Vitamin B12 |  |  |  |  |
| Vitamin D3 |  | |  |  |
| Vitamin B12 |  |  |  |  |
| Probiotics 50 billion |  |  |  |  |
| Carnivore protein powder |  |  |  |  |
| Sweet mint sugar free gum |  |  |  |  |
| Sucralose |  |  |  |  |
| Powder for making an electrolyte drink |  |  |  |  |
| Sugar-free bazooka |  |  |  |  |
| Vinali |  |  |  |  |
| MOA food supplement |  |  |  |  |
| Gum 5 peppermint without sugar |  |  |  |  |
| Biopro-Q, Omega-Q, Optimal-V, Magnical-D Vinali | | | | |
| MOA |  |  |  |  |
| Collagen protein + creatine + vitamin C |  |  |  |  |
| elite 100% whey |  |  |  |  |
| Alpha protein powder |  |  |  |  |
| Very Vanilla protein powder |  |  |  |  |
| Plant protein powder Dr. Murray |  |  |  |  |
| MCT powder |  |  |  |  |
| Protein powder iso 100 Dymatize |  |  |  |  |
| Orbit chewing gum without sugar |  |  |  |  |
| California Gold Nutrition protein powder |  |  |  |  |
| onewhey protein powder |  |  |  |  |
| HIGH 5 energy gel |  |  |  |  |
| Vega protein powder |  |  |  |  |
| Sunwarrior classic plus vanilla flavored protein powder | | | | |
| Pure vanilla extract |  |  |  |  |
| Sweetengo kohli sweetener |  |  |  |  |
| Spirulina, Chlorella, Moringa combined powder, aloe vera | | | | |
| Zone perfect |  |  |  |  |
| Magazinium supplement Now 400 mg |  |  |  |  |
| Gu tri-berry energy gel |  |  |  |  |
| Magnesium nutritional supplement |  |  |  |  |
| flordix |  |  |  |  |
| MCT oil supplement |  |  |  |  |
| Mars hi protein |  |  |  |  |
| Go energy + caffeine |  |  |  |  |
| Creatine |  |  |  |  |

**Table S3: List of popular Foods.**

| **Popular Foods: Items that accounted for the highest percentage of calories logged by the participants** |
| --- |
| Granola |
| French fries |
| Hamburger |
| Cottage cheese |
| Ice cream |
| Wholemeal Bread |
| Chicken breast |
| Schnitzel |
| Tahini |
| Chocolate cake |
| Beer |
| Lentils |
| Dates |
| Milk |
| Walnuts |
| emmer wheat bread |
| Olive oil |
| Omelette |
| Watermelon |
| Pullet |
| Burekas |
| Almonds |
| Cookies |
| Avocado |
| Salmon |
| Beef |
| Pita |
| Canned Tuna Fish |
| Chicken legs |
| Chocolate |
| Natural Yogurt |
| Apple |
| Egg |
| Potatoes |
| Cappuccino |
| Vegetable Salad |
| Cashew |
| Coffee |
| Cheesecake |
| Pasta |
| Yellow Cheese |
| Banana |
| Hummus Salad |
| Pizza |
| Rye Bread |
| Roll |
| Wine |
| Rice |

**Table S4: List of variables in the model aimed to predict baseline BMI and weight change in regression analysis**

| **List of features: Items that accounted in the model to predict baseline BMI and weight changes** | | |
| --- | --- | --- |
| Age | Fish | Watermelon |
| Beef | French fries | Whole meal Bread |
| Hummus Salad | Fruits | Wine |
| Natural Yogurt | Gender | Yellow Cheese |
| Pizza | Granola | Watermelon |
| Alcoholic Drinks | Hamburger | Whole meal Bread |
| Almonds | Hard cheese | Wine |
| Apple | Have back pain | Yellow Cheese |
| Avocado | Ice cream | *for weight change we added weight at baseline. |
| Banana | Industrialized vegetarian food |  |
| Beef products | Lentils |  |
| Beer | Low calories and diet drinks |  |
| Bread | Mean daily energy |  |
| Bread whole-wheat | Med Oil and fats |  |
| Burekas | Milk |  |
| Canned Tuna Fish | Olive oil |  |
| Canned veg and fruits | Omelette |  |
| Cappuccino | Pasta |  |
| Cashew | Pasta |  |
| Cereals | Physical activity moderate (days a week) |  |
| Cheesecake | Physical activity vigorous (days a week) |  |
| Chicken breast | Pita |  |
| Chicken legs | Potatoes |  |
| Chocolate | Pullet |  |
| Chocolate cake | RDS score |  |
| Coffee | Rice |  |
| Cookies | Roll |  |
| Deep fried foods | Rye Bread |  |
| Depressed hopeless | Salmon |  |
| Drinks | Schnitzel |  |
| Education | Shemmer wheat bread |  |
| Education complete age | Smoke tobacco (currently) |  |
| Egg | Tahini |  |
| Eggs | Total income |  |
| Employment | Vegetable Salad |  |
| English cake | Walking 10min (days a week) |  |
| Fast Foods | Walnuts |  |

**Table S5: Daily macro- and micronutrient intake by gender and BMI categories at baseline visit.**

|  | **WOMEN** | | | | **MEN** | | | |
| --- | --- | --- | --- | --- | --- | --- | --- | --- |
|  | **Normal** | **Overweight** | **Obesity** | ***p*** | **Normal** | **Overweight** | **Obesity** | ***p*** |
| N | N=2277 | N=1560 | N=705 |  | N=1510 | N=1827 | N=669 |  |
| Daily calories (Kcal) ± SD | 1504 ± 276 | 1487 ± 271 | 1511 ± 293 | 0.099 | 1831 ± 318 | 1771 ± 315 | 1762 ± 324 | 3.8E-08^ab^ |
| **Daily carbohydrates Intake** | | | | | | | | |
| Total intake (g) ± SD | 151.7 ± 41.3 | 148.5 ± 40.3 | 149.9 ± 42.1 | 0.027^a^ | 182.4 ± 51.7 | 173.1 ± 50.1 | 169.6 ± 48.9 | 1.2E-10^ab^ |
| Percent of daily calories | 40.4 | 39.8 | 39.6 | 0.008^ab^ | 39.9 | 39.0 | 38.4 | 2.7E-07^abc^ |
| Daily fiber intake  (g/1000 kcal) ± SD | 14.2 ± 4.8 | 13.6 ± 3.9 | 13.1 ± 3.7 | 8.4E-07^abc^ | 13.1 ± 4.6 | 12.2 ± 4.0 | 12.1 ± 4.2 | 1.2E-09 ^ab^ |
| **Daily protein intake** | | | | | | | | |
| Total intake (g) ± SD | 65.3 ± 16.7 | 67.9 ± 16.1 | 70.6 ± 15.9 | 4.9E-16 ^abc^ | 82.2 ± 23.1 | 83.6 ± 21.5 | 86.9 ± 21.0 | 4.0E-08 ^abc^ |
| Percent of daily calories | 17.5 | 18.4 | 18.9 | 1.0E-24^abc^ | 18.0 | 19.0 | 20.0 | 2.3E-26^abc^ |
| **Daily fat intake** | | | | | | | | |
| Total intake (g) ± SD | 64.2 ± 17.7 | 63.0 ± 15.8 | 64.6 ± 17.3 | 0.152 | 76.9 ± 21.1 | 74.1 ± 19.4 | 74.2 ± 18.9 | 0.002^ab^ |
| Percent of daily calories | 38.3 | 38.0 | 38.3 | 0.960 | 37.7 | 37.6 | 37.8 | 0.146 |
| Total daily MUFA (g) ± SD | 19.1 ± 6.8 | 18.6 ± 5.9 | 19.1 ± 6.2 | 0.158 | 22.5 ± 8.5 | 21.3 ± 7.4 | 21.2 ± 6.8 | 0.005^ab^ |
| Total daily PUFA (g) ± SD | 10.7 ± 4.4 | 10.2 ± 3.7 | 10.4 ± 3.6 | 0.005^a^ | 12.7 ± 5.6 | 11.6 ± 4.8 | 11.6 ± 4.5 | 4.8E-08^ab^ |
| Saturated fat- percent of daily calories | 8.9 | 9.2 | 9.6 | 6.1E-12^abc^ | 8.6 | 9.0 | 9.2 | 9.0E-11^abc^ |
| **Micronutrients** | | | | | | | | |
| Calcium (mg) ± SD | 470.7 ± 190.8 | 472.7 ± 175.7 | 492.8 ± 172.5 | 7.7E-04^bc^ | 503.4 ± 201.6 | 492.3 ± 189.0 | 502.0 ± 207.1 | 0.548 |
| Magnesium (mg) ± SD | 200.2 ± 73.1 | 190.5 ± 58.2 | 190.6 ± 54.9 | 6.3E-05^ab^ | 232.5 ± 81.9 | 214.4 ± 69.6 | 211.6 ± 66.1 | 1.3E-11^ab^ |
| Phosphorus (mg) ± SD | 758.9 ± 209.3 | 774.3 ± 201.5 | 811.5 ± 203.8 | 2.5E-09^abc^ | 895.9 ± 248.1 | 899.3 ± 237.9 | 931.8 ± 244.3 | 0.002^bc^ |
| Potassium (mg) ± SD | 1904.8 ± 595.1 | 1887.7 ± 560.2 | 1885.0 ± 507.5 | 0.872 | 2126.5 ± 666.0 | 2062.7 ± 628.7 | 2098.8 ± 644.7 | 0.010^a^ |
| Sodium (mg) ± SD | 2260.0 ± 619.9 | 2334.0 ± 579.9 | 2445.7 ± 688.9 | 1.4E-11^abc^ | 2750.8 ± 706.5 | 2821.0 ± 681.2 | 3010.0 ± 1052.0 | 3.8E-10^abc^ |
| Caffeine (mg) ± SD | 47.9 ± 120.2 | 54.7 ± 130.2 | 53.5 ± 110.5 | 1.3E-05^ab^ | 29.6 ± 91.4 | 35.9 ± 95.4 | 40.9 ± 108.1 | 3.5E-07^ab^ |
| Cholesterol (mg) ± SD | 215.3 ± 108.2 | 237.7 ± 110.1 | 258.5 ± 113.3 | 1.7E-23^abc^ | 262.8 ± 147.5 | 281.1 ± 134.4 | 299.2 ± 122.0 | 2.1E-14^abc^ |
| Iron (mg) ± SD | 7.53 ± 4.59 | 7.48 ± 6.82 | 8.18 ± 16.47 | 0.031^a^ | 9.56 ± 11.15 | 9.08 ± 10.47 | 8.86 ± 5.44 | 4.1E-05^ab^ |
| Niacin (mg) ± SD | 11.1 ± 3.9 | 11.5 ± 3.7 | 12.3 ± 3.9 | 9.3E-13^abc^ | 14.3 ± 5.0 | 15.1 ± 5.3 | 15.9 ± 5.2 | 1.7E-12^abc^ |
| Riboflavin (mg) ± SD | 0.94 ± 0.31 | 0.97 ± 0.30 | 1.00 ± 0.30 | 2.1E-08^abc^ | 1.06 ± 0.36 | 1.07 ± 0.34 | 1.10 ± 0.36 | 0.004^bc^ |
| Thiamin (mg) ± SD | 0.69 ± 0.22 | 0.67 ± 0.19 | 0.69 ± 0.20 | 0.057 | 0.82 ± 0.26 | 0.77 ± 0.23 | 0.79 ± 0.25 | 1.8E-06^ab^ |
| Vitamin A (ug) ± SD | 521.7 ± 273.6 | 511.4 ± 249.2 | 516.2 ± 274.1 | 0.897 | 528.6 ± 307.9 | 511.3 ± 307.9 | 530.5 ± 371.5 | 0.080 |
| Vitamin B12 (ug) ± SD | 2.53 ± 1.78 | 2.76 ± 1.77 | 2.98 ± 1.68 | 2.0E-16^abc^ | 3.15 ± 2.49 | 3.41 ± 2.44 | 3.64 ± 2.57 | 2.5E-11^abc^ |
| Vitamin B6 (mg) ± SD | 1.36 ± 1.60 | 1.29 ± 0.65 | 1.33 ± 0.67 | 0.011^bc^ | 1.66 ± 1.69 | 1.59 ± 1.18 | 1.60 ± 0.55 | 0.112 |
| Vitamin C (mg) ± SD | 90.8 ± 55.2 | 88.2 ± 47.3 | 84.9 ± 41.6 | 0.393 | 95.1 ± 56.7 | 89.1 ± 61.2 | 91.2 ± 57.3 | 2.0E-04^ab^ |
| Vitamin E (mg) ± SD | 6.17 ± 2.78 | 5.89 ± 2.28 | 5.84 ± 2.00 | 0.037^a^ | 6.79 ± 3.13 | 6.30 ± 2.86 | 6.14 ± 2.36 | 3.6E-07^ab^ |
| Zinc (mg) ± SD | 5.95 ± 3.31 | 6.08 ± 3.11 | 6.40 ± 3.27 | 1.8E-07^abc^ | 7.49 ± 3.46 | 7.63 ± 3.55 | 8.15 ± 6.59 | 2.7E-05^bc^ |

BMI- body mass index. Normal weight- BMI 18.5-25, Overweight- BMI 25-30, Obesity BMI≥30. Underweight -BMI<18.5 were included in the Normal weight group due to small sample size and near normal BMI.

In post hoc analysis there was a significant difference between: a- normal and overweight b- normal-weight and obesity c- overweight and obesity categories.

**Table S6: Beta-coefficients, Confidence intervals, and P values for regression analyses predicting baseline BMI in women.** (per kcal)

|  | CI Low | CI High | Beta | p- value |
| --- | --- | --- | --- | --- |
| age | 0.081502 | 0.117738 | 0.09962 | 9.18E-27 |
| walking_10min_days_a_week | -0.15949 | -0.03543 | -0.09746 | 0.002081 |
| physical_activity_maderate_days_a_week | -0.28021 | -0.1447 | -0.21246 | 8.56E-10 |
| physical_activity_vigorous_days_a_week | -0.42193 | -0.06323 | -0.24258 | 0.008038 |
| education | -0.3657 | -0.15118 | -0.25844 | 2.39E-06 |
| education_complete_age | 0.002692 | 0.036062 | 0.019377 | 0.022846 |
| rds_score | 0.062777 | 0.263857 | 0.163317 | 0.001459 |
| have_back_pain | 0.304397 | 0.934908 | 0.619653 | 0.000118 |
| mean_daily_energy | 0.000893 | 0.004723 | 0.002808 | 0.00406 |
| Canned Tuna Fish | 0.007208 | 0.023079 | 0.015143 | 0.000185 |
| Pullet | -0.01456 | -0.00129 | -0.00792 | 0.019328 |
| Milk | 0.001182 | 0.01113 | 0.006156 | 0.015299 |
| Wine | -0.01756 | -0.00034 | -0.00895 | 0.04158 |
| Roll | -0.0111 | -0.00042 | -0.00576 | 0.034487 |
| Yellow Cheese | 0.004094 | 0.01627 | 0.010182 | 0.001051 |
| Alcoholic Drinks | -0.01398 | -0.00066 | -0.00732 | 0.031248 |
| Cereals | -0.01229 | -0.00407 | -0.00818 | 9.58E-05 |
| Fast Foods | 0.002148 | 0.015263 | 0.008706 | 0.009278 |
| Fruits | -0.00786 | -0.00137 | -0.00462 | 0.005268 |
| Nuts seeds and products | -0.01001 | -0.00423 | -0.00712 | 1.44E-06 |
| Pasta Grains and Side dishes | -0.00554 | -0.00018 | -0.00286 | 0.036145 |
| Poultry and its products | 0.000191 | 0.006959 | 0.003575 | 0.038406 |
| Processed meat products | 0.004693 | 0.016011 | 0.010352 | 0.000339 |
| Soups and sauces | 0.002266 | 0.017952 | 0.010109 | 0.011544 |
| Fruit juices and soft-drinks | -0.0135 | -0.0031 | -0.0083 | 0.001751 |
| sweets | -0.00517 | -0.00046 | -0.00281 | 0.019208 |

**Table S7: Beta-coefficients, Confidence intervals, and P values for regression analyses predicting baseline BMI in men.** (per kcal)

|  | CI low | CI high | Beta | p-value |
| --- | --- | --- | --- | --- |
| age | 0.059844 | 0.092954 | 0.076399 | 2.25E-19 |
| physical_activity_maderate_days_a_week | -0.25852 | -0.13348 | -0.196 | 8.71E-10 |
| physical_activity_vigorous_days_a_week | -0.50879 | -0.25248 | -0.38064 | 6.24E-09 |
| education | -0.19526 | -0.00904 | -0.10215 | 0.03154 |
| education_complete_age | 0.007072 | 0.045733 | 0.026403 | 0.007441 |
| Burekas | 0.00079 | 0.011632 | 0.006211 | 0.024739 |
| Chocolate | 0.000161 | 0.010773 | 0.005467 | 0.043439 |
| Hamburger | -0.01041 | -0.00258 | -0.00649 | 0.001152 |
| Cottage cheese | 0.000297 | 0.009561 | 0.004929 | 0.037016 |
| Apple | 0.00441 | 0.016008 | 0.010209 | 0.000563 |
| Pasta | -0.00918 | -0.00136 | -0.00527 | 0.00825 |
| Egg | -0.01982 | -0.00559 | -0.0127 | 0.000472 |
| Yellow Cheese | 0.000368 | 0.009212 | 0.00479 | 0.033756 |
| Banana | -0.01008 | -0.00069 | -0.00539 | 0.024581 |
| Omelette | -0.01981 | -0.00759 | -0.0137 | 1.12E-05 |
| Pita | -0.00505 | -1.77E-05 | -0.00253 | 0.048408 |
| Rice | -0.0069 | -0.00095 | -0.00392 | 0.009662 |
| Beef veal lamb and other meat products | 0.001712 | 0.00532 | 0.003516 | 0.000135 |
| Canned veg. and fruits | 0.000637 | 0.010549 | 0.005593 | 0.026997 |
| Cereals | -0.00967 | -0.00388 | -0.00678 | 4.70E-06 |
| Eggs and their products | 0.005679 | 0.016675 | 0.011177 | 6.86E-05 |
| Nuts seeds and products | -0.00584 | -0.00176 | -0.0038 | 0.00026 |
| Oils and fats | -0.00742 | -0.0019 | -0.00466 | 0.000929 |
| Poultry and its products | 0.001405 | 0.005802 | 0.003604 | 0.001321 |
| Processed meat products | 0.00082 | 0.006992 | 0.003906 | 0.013119 |
| Vegetables | 0.000537 | 0.005042 | 0.002789 | 0.015212 |
| Fruit juices and soft-drinks | -0.00687 | -0.00126 | -0.00407 | 0.004499 |
| sweets | -0.00327 | -0.00016 | -0.00171 | 0.030616 |

**Table S8: Anthropometric and clinical measurements by gender and weight difference at two-year follow-up.**

|  | **WOMEN** | | | | **MEN** | | | |
| --- | --- | --- | --- | --- | --- | --- | --- | --- |
|  | **No weight change** | **Weight loss** | **Weight gain** | ***p*** | **No weight change** | **Weight loss** | **Weight gain** | ***p*** |
|  | **N=338** | **N=162** | **N=157** |  | **N=384** | **N=104** | **N=111** |  |
| Weight change (kg) ± SD | 0.01 ± 0.7 | -6.7 ± 3.5 | 5.7 ± 2.3 |  | 0.04 ± 0.9 | -7.9 ± 3.6 | 6.0 ± 2.5 |  |
| BMI (kg/m^2^) ± SD baseline | 25.0 ± 4.3 | 27.7 ± 4.6 | 25.4 ± 4.0 | <0.001^ac^ | 25.7 ± 3.3 | 27.9 ± 4.3 | 25.4 ± 3.7 | <0.001^ac^ |
| Age ± SD | 56.1 ± 8.2 | 55.2 ± 7.8 | 53.5 ± 7.5 | 0.003^ab^ | 54.9 ± 8.3 | 55.6 ± 7.9 | 52.0 ± 7.4 | 0.001^bc^ |
| Monthly household income | | | |  |  | | |  |
| 6-11k (%) | 29 (8.6) | 13 (8.0) | 17 (10.8) | 0.918 | 10 (2.6) | 3 (2.9) | 5 (4.5) | 0.909 |
| 11-15K (%) | 35 (10.4) | 18 (11.1) | 13 (8.3) |  | 28 (7.3) | 6 (5.8) | 8 (7.2) |  |
| 15-21k (%) | 69(20.4) | 35 (21.6) | 31 (19.7) |  | 77 (20.1) | 21 (20.2) | 16 (14.4) |  |
| 21-36K (%) | 126 (37.3) | 66 (40.7) | 60 (38.2) |  | 184 (47.9) | 48 (46.2) | 55 (49.5) |  |
| >36K (%) |  |  |  |  |  |  |  |  |
| Unknown* (%) | 79 (23) | 30 (18.5) | 36 (22.9) |  | 85 (22.1) | 26 (25.0) | 27 (24.3) |  |
| Change in systolic BP (mmHg) ± SD | -0.81 ± 13.6 | -4.0 ± 13.7 | 1.9 ± 13.8 | <000.1^ac^ | -1.77 ± 11.9 | -7.5 ± 13.2 | 2.9 ± 13.5 | <0.001^abc^ |
| Change in diastolic BP (mmHg) ± SD | -1.75 ± 8.3 | -3.2 ± 8.2 | -0.2 ± 8.9 | 0.003^ac^ | -1.0 ± 8.2 | -5.8 ± 8.2 | 0.1 ± 8.5 | 0.003^abc^ |

No weight change defined <2% change at follow-up. Weight loss ≥5% weight reduction at follow-up. Weight gain ≥5% weight gain at follow-up. In post hoc analysis there was a significant difference between: a- no change and weight loss b- no change and weight gain c- weight loss and weight gain. *answered “don’t know” or “prefer not to say”

**Table S9: Comparing first visit and follow-up cohort demographics.**

|  | **First visit** | **Follow-up visit** | **p-value** |
| --- | --- | --- | --- |
| **Age** mean ± SD | 51.73 ± 7.79 | 52.61 ± 8.10 | <0.001 |
| **Female** % | 53.1 | 53.0 | 0.884 |
| **BMI** mean ± SD | 26.05 ± 4.12 | 25.87 ± 4.11 | 0.061 |
| **Mild physical activity*** days. mean ± SD | 4.28 ± 2.05 | 4.20 ± 2.15 | 0.136 |

BMI- body mass index. *Physical activity- days a week – walking more than 10 minutes

**Table S10: Daily macro- and micronutrient intake by gender and weight and difference at two-year follow-up.**

|  | **WOMEN** | | | | **MEN** | | | |
| --- | --- | --- | --- | --- | --- | --- | --- | --- |
|  | **No weight change** | **Weight loss** | **Weight gain** | ***p*** | **No weight change** | **Weight loss** | **Weight gain** | ***p*** |
|  | **N=338** | **N=162** | **N=157** |  | **N=384** | **N=104** | **N=111** |  |
| Daily calories (Kcal) ± SD baseline | 1552 ± 308 | 1542 ± 313 | 1546 ± 328 | 0.981 | 1859 ± 331 | 1813 ± 316 | 1867 ± 307 | 0.453 |
| Daily calories change (Kcal) ± SD | -160 ± 248 | -206 ± 288 | -115 ± 279 | 0.012^ac^ | -205 ± 312 | -160 ± 330 | -229 ± 295 | 0.344 |
| Change in daily carbohydrates Intake | | | | | | | | |
| Total intake (g) ± SD | -20.0 ± 32.3 | -29.8 ± 45.7 | -16.5 ± 35.2 | 0.019^ac^ | -25.6 ± 41.0 | -30.8 ± 51.7 | -21.9 ± 51.9 | 0.532 |
| Percent of daily calories | -0.9 ± 5.7 | -2.3 ± 9.2 | -1.3 ± 6.4 | 0.380 | -1.3 ± 6.4 | -4.1 ± 9.2 | -0.7 ± 8.3 | 0.035^a^ |
| Daily fiber intake  (g/1000 kcal) ± SD | -0.3 ± 3.6 | -0.4 ± 4.2 | -0.8 ± 4.4 | 0.628 | -0.3 ± 3.6 | -0.4 ± 4.2 | -0.8 ± 4.4 | 0.031^c^ |
| Change in daily protein intake | | | | | | | | |
| Total intake (g) ± SD | -6.2 ± 15.2 | -6.9 ± 17.1 | -0.6 ± 16.7 | 0.002^bc^ | -7.3 ± 18.4 | -4.0 ± 22.0 | -9.6 ± 19.8 | 0.158 |
| Percent of daily calories | 0.3 ± 3.3 | 0.6 ± 4.4 | 1.2 ± 4.2 | 0.072 | 0.5 ± 3.4 | 1.0 ± 4.0 | 0.5 ± 4.0 | 0.718 |
| Change in daily fat intake | | | | | | | | |
| Total intake (g) ± SD | -5.1 ± 14.7 | -6.3 ± 17.2 | -4.5 ± 17.1 | 0.211 | -6.6 ± 17.8 | -1.4 ± 23.3 | -9.7 ± 18.9 | 0.020^ac^ |
| Percent of daily calories | 0.9 ± 5.5 | 1.5 ± 7.4 | 0.4 ± 5.7 | 0.707 | 1.1 ± 5.5 | 3.3 ± 8.7 | 0.4 ± 6.6 | 0.107 |
| Total daily MUFA (g) ± SD | -1.8 ± 6.4 | -1.1 ± 6.9 | -2.1 ± 7 | 0.770 | -2 ± 7.5 | -1.2 ± 8.9 | -4 ± 8.3 | 0.159 |
| Total daily PUFA (g) ± SD | -1.3 ± 4.3 | -1.7 ± 4.5 | -1.8 ± 4.9 | 0.399 | -1.8 ± 5.5 | -1 ± 6.5 | -2.6 ± 5.5 | 0.329 |
| Saturated fat- percent of daily calories | 0.1 ± 2.5 | 0.5 ± 2.8 | 0.0 ± 3.0 | 0.248 | 0.2 ± 2.5 | 0.3 ± 3.7 | 0.1 ± 2.8 | 0.533 |
| Micronutrients (change in daily intake) | | | | | | | | |
| Calcium (mg) ± SD | -68.9 ± 167 | -71.8 ± 165.5 | -35.8 ± 197.6 | 0.038^bc^ | -51.8 ± 191.7 | -19 ± 195.1 | -45.7 ± 269.2 | 0.098 |
| Magnesium (mg) ± SD | -32.9 ± 53.7 | -35.5 ± 53.8 | -35.3 ± 67.6 | 0.690 | -39.3 ± 71.6 | -18.1 ± 78.8 | -51.4 ± 72.6 | 0.009^ac^ |
| Phosphorus (mg) ± SD | -114.1 ± 180.1 | -117.7 ± 199.4 | -75.3 ± 208.7 | 0.180 | -119.7 ± 265.3 | -93.5 ± 257.8 | -162.3 ± 221.2 | 0.094 |
| Potassium (mg) ± SD | -297.8 ± 486.3 | -324.4 ± 474.9 | -252.8 ± 522.8 | 0.683 | -312 ± 656.3 | -138.6 ± 680.7 | -492.4 ± 577.8 | <0.001^abc^ |
| Sodium (mg) ± SD | -195.9 ± 622.3 | -218.9 ± 606.1 | -135.7 ± 604.9 | 0.494 | -204.1 ± 883.8 | -279.3 ± 676 | -282.5 ± 859 | 0.301 |
| Caffeine (mg) ± SD | -4.7 ± 103.4 | -11.4 ± 109.2 | -12.7 ± 89.7 | 0.504 | -4.2 ± 69.9 | -17.8 ± 76.9 | -1.2 ± 71.4 | 0.052 |
| Cholesterol (mg) ± SD | -9.3 ± 105.3 | -4.7 ± 135.3 | 1 ± 121.2 | 0.321 | -22.5 ± 114.9 | -2.7 ± 140.2 | -11.3 ± 159.2 | 0.636 |
| Iron (mg) ± SD | -1 ± 3 | -0.3 ± 9.3 | -1 ± 2.5 | 0.851 | -1.1 ± 6.1 | -0.6 ± 3.5 | -6.7 ± 58.6 | 0.053 |
| Niacin (mg) ± SD | -1.6 ± 3.7 | -1.6 ± 3.8 | -0.5 ± 4.4 | 0.037^bc^ | -1.8 ± 4.9 | -1.9 ± 4.9 | -3 ± 5.6 | 0.041^b^ |
| Riboflavin (mg) ± SD | -0.1 ± 0.3 | -0.1 ± 0.4 | -0.1 ± 0.3 | 0.640 | -0.1 ± 0.4 | -0.1 ± 0.3 | -0.2 ± 0.4 | 0.233 |
| Thiamin (mg) ± SD | -0.1 ± 0.2 | -0.1 ± 0.2 | -0.1 ± 0.2 | 0.032^ac^ | -0.1 ± 0.3 | -0.1 ± 0.3 | -0.1 ± 0.3 | 0.998 |
| Vitamin A (ug) ± SD | -89.2 ± 365.3 | -30.9 ± 317.5 | -62.8 ± 309.2 | 0.406 | -79.8 ± 458.7 | 45.9 ± 268.2 | -175 ± 655 | <0.001^abc^ |
| Vitamin B12 (ug) ± SD | -0.5 ± 3 | 0 ± 1.9 | -0.1 ± 1.8 | 0.033^b^ | -0.3 ± 3.2 | -0.3 ± 1.6 | -0.9 ± 5.1 | 0.813 |
| Vitamin B6 (mg) ± SD | -0.1 ± 3.2 | -0.2 ± 0.4 | -0.1 ± 0.8 | 0.052 | -0.3 ± 1.5 | 0.2 ± 3.1 | -0.4 ± 0.5 | 0.001^bc^ |
| Vitamin C (mg) ± SD | -15.6 ± 56.4 | -10.2 ± 51.7 | -12.9 ± 48.2 | 0.574 | -9.8 ± 72.1 | 10.9 ± 67.5 | -20.8 ± 52 | <0.001^ac^ |
| Vitamin E (mg) ± SD | -0.6 ± 3.2 | -0.7 ± 2.5 | -1 ± 3 | 0.162 | -0.8 ± 3 | 0 ± 4 | -0.8 ± 3.3 | 0.010^ac^ |
| Zinc (mg) ± SD | -0.8 ± 2.1 | -0.7 ± 2.3 | 0.3 ± 7.5 | 0.538 | -0.9 ± 3.5 | -1 ± 3.6 | -1.8 ± 4.8 | 0.128 |

No weight change defined <2% change at follow-up. Weight loss ≥5% weight reduction at follow-up. Weight gain ≥5% weight gain at follow-up.

In post hoc analysis there was a significant difference between: a- no change and weight loss b- no change and weight gain c- weight loss and weight gain

**Table S11: Beta-coefficients, Confidence intervals, and P values for regression analyses predicting weight change in women at follow-up.**

|  | CI low | CI High | Beta | Calories per portion | Effect per portion | P value |
| --- | --- | --- | --- | --- | --- | --- |
| Age (y) | -0.06973 | -0.00671 | -0.03822 |  |  | 0.010044 |
| Weight at Baseline (kg) | -0.07069 | -0.02896 | -0.04982 |  |  | 0.00026 |
| Caloric Diff | 0.00122 | 0.007757 | 0.004489 | 100 | 0.448850441 | 0.033902 |
| Cashew (PF) | -0.02381 | -0.00369 | -0.01375 | 250 | -3.437663666 | 0.013302 |
| Rye Bread (PF) | 0.000745 | 0.022836 | 0.01179 | 80 | 0.943238453 | 0.001553 |
| Apple (PF) | -0.02842 | -0.00175 | -0.01509 | 80 | -1.206884253 | 0.003757 |
| Pita (PF) | 0.002706 | 0.018568 | 0.010637 | 200 | 2.127395589 | 0.027891 |
| Pizza (PF) | 0.001666 | 0.01321 | 0.007438 | 300 | 2.231427727 | 0.047909 |
| Rice (PF) | 6.12E-05 | 0.014509 | 0.007285 | 220 | 1.602708848 | 0.04635 |
| Vegetables (FC) | -0.01133 | -0.00031 | -0.00582 | 50 | -0.29114975 | 0.028258 |
| Sweets (FC) | -0.00871 | -0.00061 | -0.00466 | 100 | -0.466461567 | 0.04867 |

|  | CI low | CI High | Beta | Calories per portion | Effect per portion | P value |
| --- | --- | --- | --- | --- | --- | --- |
| Age (y) | -0.09265 | -0.02653 | -0.05959 |  |  | 0.035846 |
| Weight at Baseline (kg) | -0.07896 | -0.03676 | -0.05786 |  |  | 0.000218 |
| Egg (PF) | -0.02887 | -0.00175 | -0.01531 | 80 | -1.225046852 | 0.004556 |
| Beef (PF) | -0.01067 | -0.00081 | -0.00574 | 265 | -1.521508505 | 0.024069 |
| Vegetable Salad (PF) | -0.01827 | -0.00259 | -0.01043 | 100 | -1.042977247 | 0.000514 |
| Rice (PF) | 0.003249 | 0.015088 | 0.009169 | 220 | 2.01715732 | 0.001102 |
| Bread, wholewheat (FC) | 0.001752 | 0.013884 | 0.007818 | 80 | 0.625401908 | 0.001225 |
| Fruits (FC) | -0.01231 | -0.00079 | -0.00655 | 80 | -0.524382252 | 0.036899 |
| Spicesandherbs (FC) | -0.06731 | -0.00644 | -0.03687 | 10 | -0.368747135 | 0.026838 |

**Table S12: Beta-coefficients, Confidence intervals, and P values for regression analyses predicting weight change in men at follow-up.**

**Tabe S13: Comparison of selected dietary nutrient intake: percentage of '10K Cohort' participants within Israeli ‘Mabat Survey’ ranges.**

|  | | **Men** | | **Women** | | |
| --- | --- | --- | --- | --- | --- | --- |
|  | **Mabat survey mean** | **Range (± 25%)** | **percent of 10k** | **Mabat survey mean** | **Range** | **percent of 10k** |
| Daily calories (Kcal) | 1712 | 1284 - 2140 | 77.0% | 1286 | 964-1670 | 62.7% |
| Daily carbohydrate intake (% of daily calories) | 0.455 | 0.341 - 0.569 | 77.7% | 0.485 | 0.364 - 0.606 | 74.4% |
| Daily protein intake (% of daily calories) | 0.189 | 0.142 - 0.236 | 76.4% | 0.177 | 0.133 - 0.221 | 78.1% |
| Daily Fat intake (% of daily calories) | 0.334 | 0.251 - 0.418 | 79.5% | 0.325 | 0.244 - 0.406 | 71.9% |
| Daily Sodium intake (mg) | 2855 | 2141 - 3568 | 68.0% | 2049 | 1536 - 2561 | 60.6% |

**Figure S1: Correlation of Food categories and popular foods.
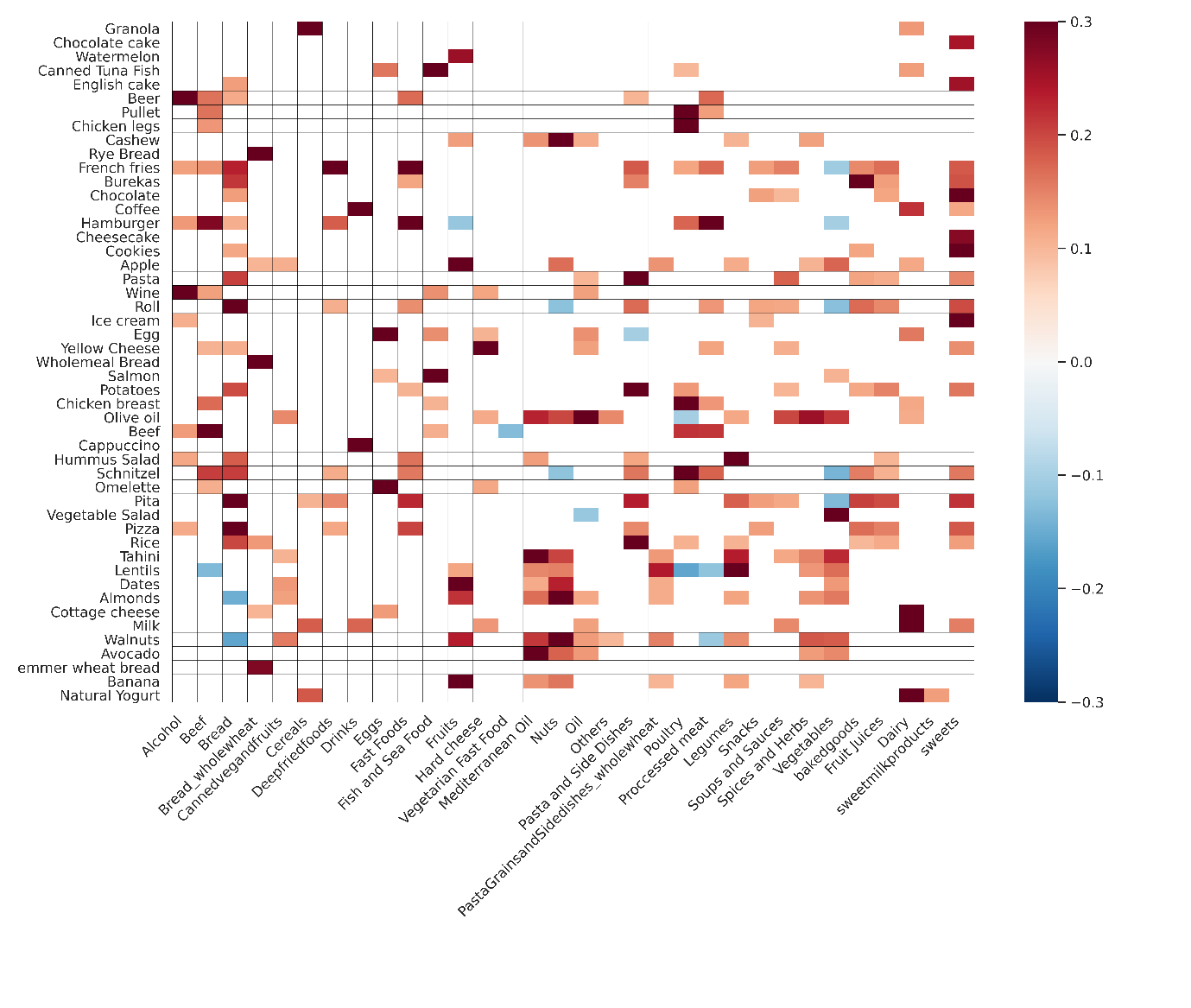
**
